# Genotype imputation and polygenic score estimation in northwestern Russian population

**DOI:** 10.1371/journal.pone.0269434

**Published:** 2022-06-28

**Authors:** Nikita Kolosov, Valeriia Rezapova, Oxana Rotar, Alexander Loboda, Olga Freylikhman, Olesya Melnik, Alexey Sergushichev, Christine Stevens, Trudy Voortman, Anna Kostareva, Alexandra Konradi, Mark J. Daly, Mykyta Artomov

**Affiliations:** 1 Almazov National Medical Research Centre, Saint-Petersburg, Russia; 2 ITMO University, Saint-Petersburg, Russia; 3 Broad Institute, Cambridge, MA, United States of America; 4 Erasmus MC, University Medical Center, Rotterdam, The Netherlands; 5 Analytic and Translational Genetics Unit, Massachusetts General Hospital, Boston, MA, United States of America; 6 Division of Human Nutrition & Health, Wageningen University, Wageningen, The Netherlands; 7 Institute for Molecular Medicine Finland (FIMM), Helsinki, Finland; Illumina Inc, UNITED STATES

## Abstract

Numerous studies demonstrated the lack of transferability of polygenic score (PGS) models across populations and the problem arising from unequal presentation of ancestries across genetic studies. However, even within European ancestry there are ethnic groups that are rarely presented in genetic studies. For instance, Russians, being one of the largest, diverse, and yet understudied group in Europe. In this study, we evaluated the reliability of genotype imputation for the Russian cohort by testing several commonly used imputation reference panels (e.g. HRC, 1000G, HGDP). HRC, in comparison with two other panels, showed the most accurate results based on both imputation accuracy and allele frequency concordance between masked and imputed genotypes. We built polygenic score models based on GWAS results from the UK biobank, measured the explained phenotypic variance in the Russian cohort attributed to polygenic scores for 11 phenotypes, collected in the clinic for each participant, and finally explored the role of allele frequency discordance between the UK biobank and the study cohort in the resulting PGS performance.

## Introduction

Over the last decade, genome-wide association studies (GWAS) have discovered a substantial number of associated variants for many complex traits. Yet, the non-uniform representation of populations in genetic studies considerably limits the applicability of GWAS-based resources for individual risk prediction [1–4]. For example, Martin et al. [1] showed that polygenic scores (PGS) are far more accurate for European individuals than for non-Europeans. The lack of parity in PGS accuracy occurs due to the overwhelming abundance of European-descent association studies.

However, even within European-centered GWAS data, there are significantly under-represented local populations. For example, Russians are one of the largest ethnic groups among the Europeans and, while there are numerous prior reports about genetic diversity in Russia [5–7], GWAS efforts have been quite limited. Russian descent samples have mostly been used in consortium studies as replication cohorts [8, 9] and no large-scale population-specific GWASs have been presented. Therefore, PGS models based on ancestry-specific GWAS results are yet to be defined and explored in Russian populations.

Given the absence of ancestry-matched supporting resources, other common publicly available databases (e.g. 1000G [10], HGDP [11], UKBB [12]) should be used for data preparation procedures preceding PGS calculation, such as genotype imputation and selection of appropriate GWAS summary statistics. However, the utility of reference databases has never been tested in application to the Russian population. However, understanding the power and limitations of such resources is essential for the translation of global GWAS discoveries (PGS models, risk variants, etc.) into efficient individual risk prediction for local cohorts.

Here, we present an analysis of the array-based genotyping data obtained from Russian-descent individuals. We selected an optimal genotype imputation panel based on the accuracy metrics calculated for masked genotypes and evaluated the concordance of polygenic score estimates based on UK-biobank GWAS results with clinically measured phenotypes.

## Materials and methods

An extended description of the technical pipeline and parameters used for data processing is available in the **S1 Appendix**.

### Dataset

239 DNA samples were collected from the elderly population at the Almazov National Medical Research Centre (St. Petersburg, Russia). All participants were Russians from Saint-Petersburg. Clinical information was obtained by physician specialists during the ambulatory patient visit (**S1 Table in S1 Appendix**). All participants provided their written informed consent [13].

DNA samples were genotyped using the GSA Illumina v2.0 array at Broad Institute and subjected to the quality filtering pipeline (see **S1 Appendix, Study Data Processing**). The final dataset consisted of 230 samples and 501,100 directly genotyped variants.

### Reference panels for genotype imputation

Genotype imputation in the Russian population was carried out using three reference panels: the Haplotype Reference Consortium (HRC) [14], 1000 Genome Project (1000G; Phase 3, Version 5) [10] and Human Genome Diversity Project (HGDP) [11]. The HRC, 1000G and HGDP panels included 27,165, 2,504, and 929 individuals, respectively. All three were preprocessed and filtered to meet the data formatting requirements for further imputation procedure. HGDP was additionally lifted over to GRCh37 (UCSC hg19). After the filtering there were 37,620,211 variants left for HRC, 37,522,002 for 1000G and 26,678,803 for HGDP (see **S1 Appendix, Reference Panel Processing**).

### Genotype imputation

Before the imputation, genotypes were pre-phased, strand-checked, and split into individual chromosomes (see **S1 Appendix, Pre-imputation Study Data Processing**). All imputations were performed with Beagle 5.2 [15] with the default parameters (*burnin* = 6, *iterations* = 12, *imp-segment* = 6, *ne* = 1000000). The imputation quality for each variant was measured using R^2^ (Dosage-R^2^; DR2), as given in Beagle output [15]. All variants with DR2 > = 0.8 were considered well-imputed and kept for further analysis. The threshold was chosen based on the results of testing of imputation accuracy (see **S1 Appendix, Genotype Imputation Accuracy**).

### Imputation accuracy

We tested three common measures of accuracy of imputation: concordance rate, squared correlation, and imputation quality score (IQS) [16–18].

Concordance rate (CR) was estimated as the sum of genotype probabilities for each matching genotypic class divided by the total number of genotypes [17]. Thus, the probabilistic nature of the imputation was taken into account.

The squared correlation coefficient (r^2^) was measured as the squared Pearson correlation between the directly measured genotypes and the imputed dosages. Directly measured genotypes were encoded according to the minor allele occurrence. Thus, major homozygote was encoded as 0, the minor homozygote as 2, and both heterozygotes (0|1 or 1|0) as 1. Allele dosages, taking values in the range from 0 to 2, were extracted from Beagle 5.2 output.

As an alternative to the previous two methods, we used the imputation quality score (IQS). It measures the agreement between two genotype sets using the concordance rate (P_o_), but in this case adjusted for the chance agreement (P_c_). The IQS calculation was performed using the following equation:

$$IQS=\frac{P_{o}-P_{c}}{1-P_{c}}$$

where P_o_ is the concordance rate and P_c_ is the chance agreement.

Chance agreement (P_c_) is the sum of the products of marginal frequencies that would occur if genotypes were called at random using the same marginal rates [17, 18].

### Allele frequency concordance

In this study, we used allele frequency concordance between two sets of variants as an additional measure of imputation quality.

All variants with allele frequency values differing more than 0.1, between imputed and masked dataset, or fall outside of +/- 5 log_2_ fold changes were considered discordant. All others were classified as concordant variants. In that way, the percentage of discordant variants was defined as the number of discordant variants divided by the total number of masked variants.

Additionally, to assess the discrepancy between the imputed and observed allele frequencies, we used the Mean Absolute Error (MAE), defined as follows:

$$MAE=\frac{\sum_{i=1}^{n}|O_{i}-I_{i}|}{n}$$

where n–is the total number of tested SNPs, O_i_—is the observed allele frequency, I_i_—is the imputed allele frequency.

MAE is zero when imputed frequencies totally match the real ones and it is large when there is a major discordance between two sets.

### Polygenic scores

The phenotypic variance explained by polygenic scores was evaluated according to the protocol described in Martin et al. [1]. We used UK biobank summary statistics for 11 phenotypes that were collected for the Russian cohort: body mass index (BMI), weight, height, waist circumferences, hip circumferences, diastolic blood pressure (DBP), systolic blood pressure (SBP), triglyceride (TG), total cholesterol (TC), glucose, high-density lipoprotein (HDL). PGS was computed using PLINK 1.9 [19] (**S1 Appendix, Dataset Quality Filtering for Polygenic Score Estimation**).

## Results

**Fig 1:**
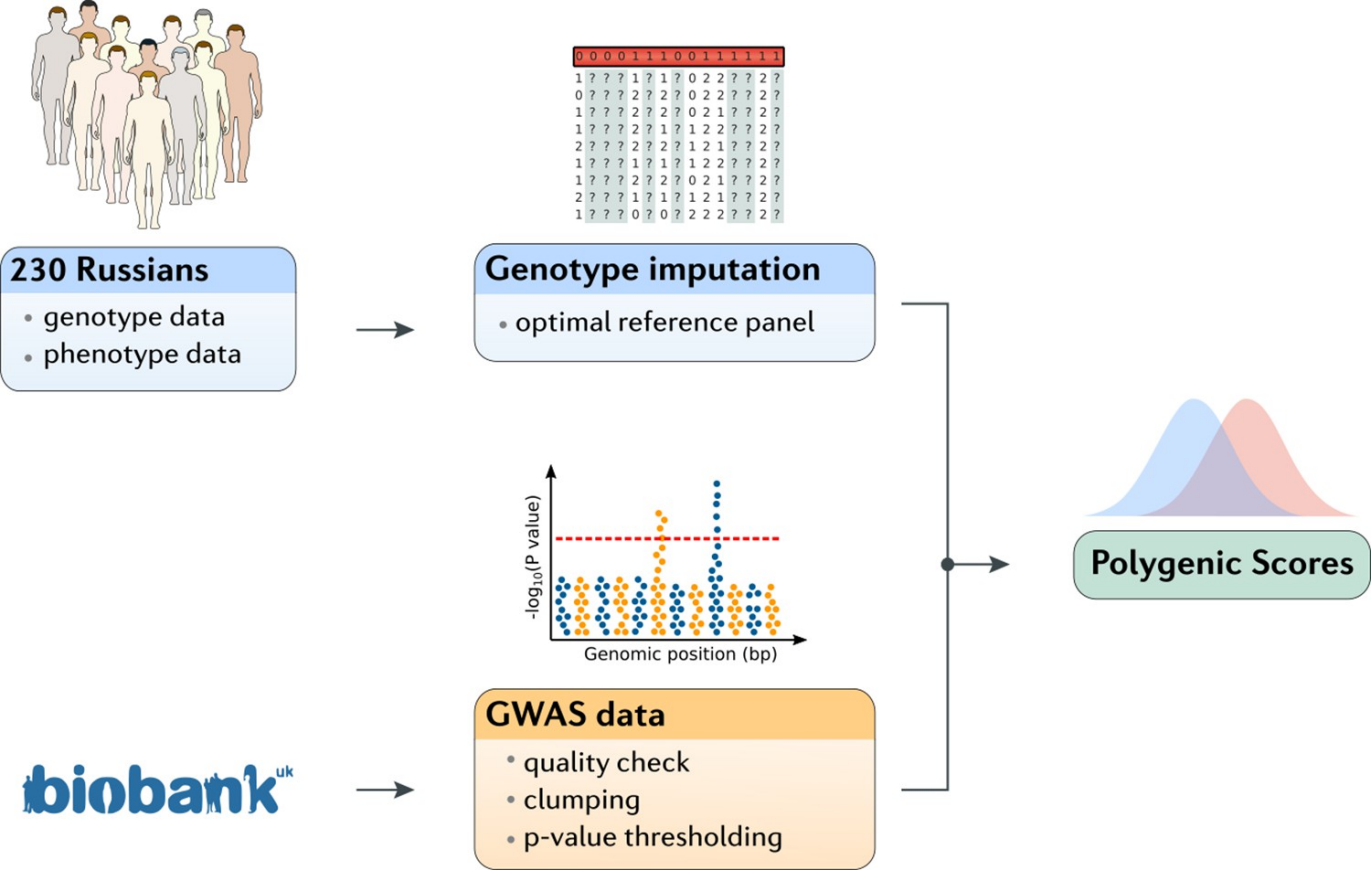
Research design. Research design scheme. 230 individuals of Russian descent were genotyped and imputation was performed using an optimal reference panel. Furthermore, UK biobank (UKBB) GWAS results were used as a reference to build optimal polygenic risk models for 11 phenotypes. Explained phenotypic variance was compared with other populations.

The genotyping data analysis protocol for the estimation of polygenic scores involves several steps that require reference data (**Fig 1**). The genotype imputation is used to increase the number of DNA variants available for analysis by using a reference whole genome sequencing panel to predict the genotypes that were not a part of the genotyping array. In the absence of population-specific resources, other common reference panels, such as HRC (Haplotype Reference Consortium), 1000G (1000 Genomes project) or HGDP (Human Genome Diversity Project), are commonly used. However, their efficiency in predicting genotypes for individuals of Russian ancestry has never been evaluated. Furthermore, the comprehensive evaluation of the imputation results is greatly impeded due to the lack of sufficient population-specific whole genome sequencing data, except several projects included a limited number of Russian-descent individuals [11, 20, 21]. This challenge makes it impossible to directly compare allele frequencies of imputed variants with unbiased whole genome sequencing data.

We overcome this issue by performing a masking experiment, where we created a study sample with a set of randomly masked variants to mimic the absence of these variants in the SNP array. We evaluated the concordance of the genotypes observed in the masked variants obtained by genotyping or imputation. Therefore, we comprehensively evaluated the reliability of the imputation using each panel for the Russian population and selected the most effective one. The latter was selected for genotype imputation in the original study data for further analysis.

Next, we calculated PGSs, tested UK-biobank GWAS utility in predicting individual polygenic scores in Russian individuals, compared explained phenotypic variance with other populations and explored the effect of allele frequency discordance between used summary statistics and study data on resulting PGS predictive ability.

Finally, we compared the applicability of the UK-biobank GWAS summary statistics for PGS estimation in central Europeans and northwestern Russians and discovered many variants whose allele frequencies in the Russian cohort considerably deviated from the UK-biobank and were significantly associated with numerous traits from the GWAS catalog [22].

### Imputation reliability for northwestern Russian cohort

We performed a validation study for imputed genotypes to evaluate the imputation accuracy for three reference panels—HRC, 1000G, HGDP. This analysis was performed with “masking” technique: before conducting the imputation, we randomly sampled 9% of variants from each chromosome in the directly genotyped data and set them aside until completion of the imputation pipeline. All sampled variants (n = 47,209) had non-zero MAF in the Russian population and were present in all used reference panels. Subsequently, we compared genotypes for these variants in genotyped and imputed data sets to calculate the accuracy scores of the imputation and the concordance of the produced allele frequencies.

First, we tested three commonly used imputation accuracy measures—concordance rate (CR), squared Pearson correlation and imputation quality score (IQS)—to compare performance of the proposed metrics (**S1-S5 Figs** in **S1 Appendix**). Our results provide further evidence that the concordance rate, in comparison with IQS and other scores studied, inflates accuracy estimates, particularly for low-frequency variants. The squared Pearson correlation was immeasurable for variants with uniform dosages, making it difficult to reliably compare accuracy for a fraction of rare variants. More specifically, some masked variants were imputed with uniform dosages, thus having zero MAFs. As a result, there was zero variation in the response variable, making the correlation coefficient between the masked and imputed dosage immeasurable. IQS was not exposed to the above biases and avoided overly permissive quality assessments for all of the frequency groups. Therefore, we used IQS as the main accuracy score for further comparison of the imputation reference panels. The observed results fit well with previous reports on other populations, suggesting that these accuracy measures perform the same regardless of the choice of the reference panel or the population studied [16, 23].

**Fig 2:**
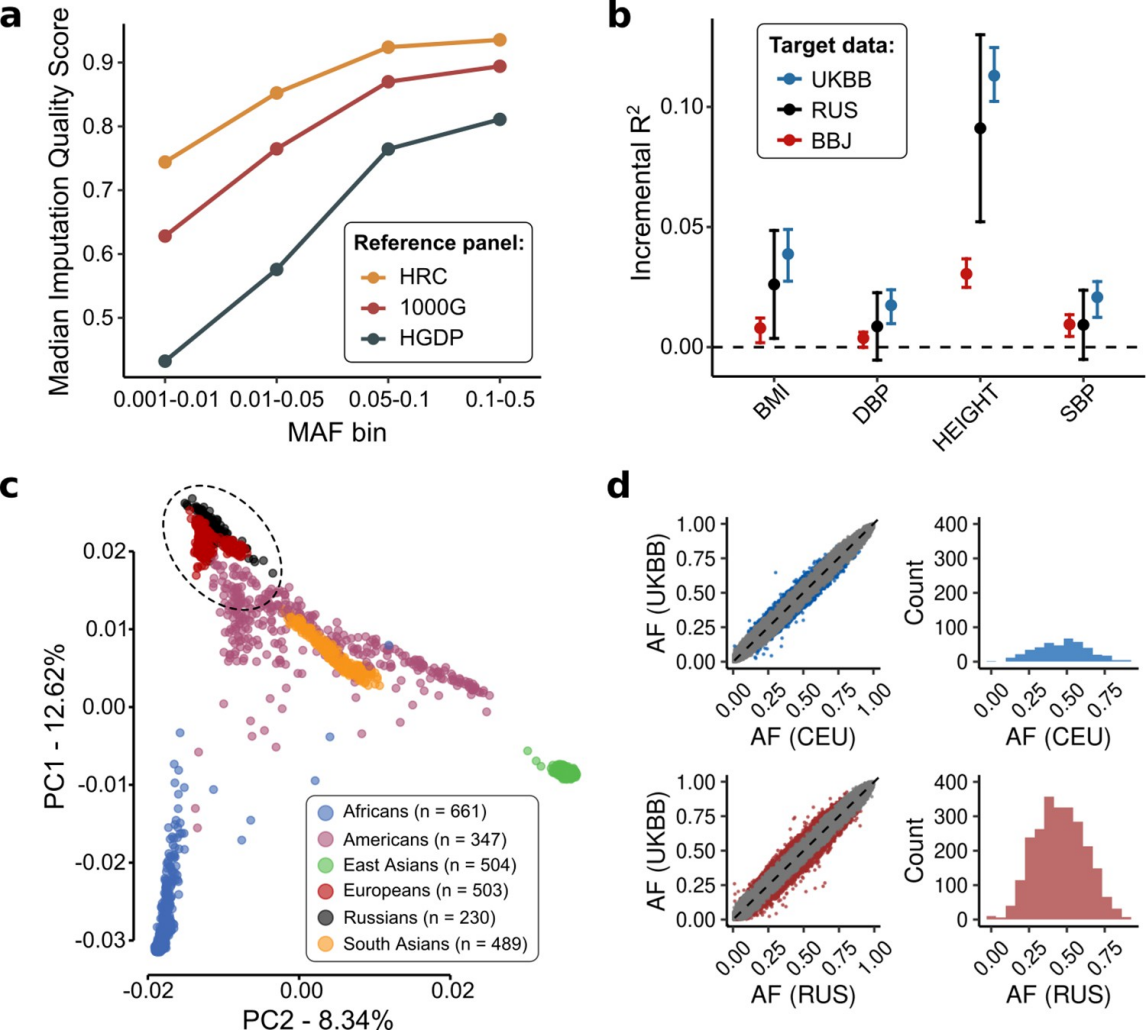
Experimental results. **(A)** Comparison of median imputation quality score for different minor allele frequency groups for HRC, 1000 Genomes and HGDP reference panels; HRC—Haplotype Reference Consortium, 1000G - 1000 Genomes project, HGDP—Human Genome Diversity Project. **(B)** Phenotypic variance explained in Japanese (BBJ), British (UKBB) and Russian (RUS) descent individuals using independent UK Biobank GWAS. **(C)** Principal Component Analysis (PCA) of 1000G, combined with Russian individuals **(D)** Concordance of allele frequencies between northwestern Russian (RUS), Central European from 1000G (CEU) and Great-Britain populations (UKBB). AF—alternative allele frequency, Grey—concordant variants, Red—discordant variants for northwestern Russians, Blue—discordant variants for Central Europeans, Dashed line—ideal concordance model, when allele frequencies of the first population totally match allele frequencies of the second population.

Further, we compared imputation reference panels based on the resulting IQS values and the concordance of imputed allele frequencies with the masked ones. HRC showed the highest IQS in comparison with other panels across different minor allele frequency (MAF) groups, especially for less common variants (**Fig 2A, S2 Table** in **S1 Appendix**). Also, the HRC reference panel showed the lowest number of discordant imputed variants (n = 607, ~1%). In contrast, 1000G had 1679 (~3%) and HGDP had 8494 (~17%) discordant variants (**S6 Fig** and **S3 Table** in **S1 Appendix**). Mean absolute error (MAE) between imputed and observed allele frequencies yielded the same qualitative outcome—HRC had the lowest error (MAE = 0.007) in comparison with 1000G (MAE = 0.011) and HGDP (MAE = 0.021). Based on these results, we showed that the HRC provides the most confident estimates of allele frequencies than 1000G and HGDP.

Taken together, we determined that, in the absence of a population-specific whole genome sequencing panel, HRC is an optimal imputation reference panel for imputing genotypes from a Russian cohort based on both imputation accuracy and allele frequency concordance between masked and imputed variants.

### Polygenic score estimation

After genotype imputation with the HRC reference panel, we estimated polygenic scores for each of the 11 phenotypes in the Russian cohort.

First, we determined variants that should be included in the PGS model using multiple *p*-value thresholds imposed on the reference UK biobank GWAS summary statistics. The 95% confidence intervals were calculated by bootstrapping with 1000 iterations. We used the nested and full models as described in Martin et al. [1] to evaluate the concordance of the polygenic score with actual phenotypes. The full linear model was given as: *phenotype ~ PGS + age + age*^*2*^ + sex + sex*age + sex*age^*2*^ *+ PC(1–10)* and the nested model contained all covariates as full, excluding *PGS*. R^2^ attributed to PGS was estimated as the difference between R^2^ of the full and the nested models. Optimal *p*-value threshold for inclusion of variants in the PGS model was selected based on the highest incremental R^2^ (**S7-S8 Figs** in **S1 Appendix**).

Further, we compared the R^2^ estimates for polygenic scores in the Russian population with the previously reported UK biobank GWAS-based R^2^ estimates for other populations [1] (**Fig 2B**). PGSs calculated based on UK biobank GWAS summary statistics resulted in the largest phenotypic variance explained for UK biobank participants. The same weights used for Russian-descent individuals led to an intermediate place of the latter between the UKBB and Biobank Japan (BBJ) individuals. Remarkably, statistically significant differences between Russian and UK populations were not found for a number of phenotypes: DBP (*p*-value = 0.90, t-test), SBP (*p*-value = 0.92, t-test), BMI (*p*-value = 0.41, t-test). This is expected given the reasonable genetic similarity between Russians and the rest of Europe and the significant difference between the Russian and Japanese populations (**Fig 2C, S9 Fig** in **S1 Appendix**).

Finally, we calculated the mean absolute error (MAE) between the UKBB allele frequencies and the study cohort for each PGS model. We explored the relationship between MAE and phenotypic variance explained by the PGS (incremental R^2^) and they appeared negatively correlated (R^2^ = 0.51, *p*-value = 0.0119; **S10 Fig** in **S1 Appendix**). Thus, demonstrating that differences in allele frequencies between UK biobank and Russian cohort, contribute to systematic bias in polygenic score estimates.

### Allele frequency concordance between UKBB and Northwestern Russians

We used allele frequency concordance between the selected population and the UK biobank as an additional measure of general resource applicability for individual risk prediction.

We compared the UK biobank participants with two populations: Central Europeans (CEU) from 1000 Genomes (N = 99) and northwestern Russian individuals (RUS) from the study dataset (N = 230). The allele frequencies for UKBB (N = 360,388) were extracted from the variant annotation file (**Data availability**). Before the concordance analysis we tested all possible sources of allele frequency discrepancies and applied different highly conservative QC filtration thresholds to check the validity of observed allele frequencies in study data and to keep only genotyped variants with confirmed frequencies (see **S1 Appendix, Allele frequencies validity check**). Altogether, we kept 379,751 variants for further analysis. All of them were presented with nonzero MAFs in all three data sources (1000G, UKBB, Study data). Variants with discordant allele frequencies were identified in the same way as it was done previously (**Materials and Methods**).

The CEU cohort showed high concordance with UKBB, demonstrating only 429 (0.11%) discordant variants, whose allele frequencies considerably deviated from the UKBB values (**Fig 2D**). In contrast, for Russian individuals, we discovered 2,436 (0.66%) discordant variants. It is noteworthy that 556 of them were significantly associated with 328 traits from the GWAS catalog (**S5 Table** in **S1 Appendix**). Interestingly, BMI, Hip/Waist and Blood protein level were the most frequent phenotypes with which these discordant variants were significantly associated, in addition to such diseases as Venous thromboembolism (rs687289, p-value = 1x10^-174^), Psoriasis (rs10484554, p-value = 4x10^-214^), Inflammatory bowel disease (rs7134599 p-value = 9x10^-32^) and Keratinocyte cancer (rs2153271, p-value = 5x10^-31^).

As a result, we showed that UKBB summary statistics is more applicable for Central Europeans than to Russian individuals. Demonstrated allele frequency discordance, potentially, limits the extent to which European-centered genomic resources could be applied for polygenic risk estimation in northwestern Russian individuals.

## Discussion

In this study, we systematically evaluated the utility of common genetic resources in application to genotype imputation and polygenic score estimation for Russian-descent individuals.

We overcame the absence of ancestry-specific allele frequency reference databases needed for imputation accuracy estimation by masking some of the genotyped variants from the initial study data and, subsequently, using them as a ground truth for validation. Expectedly, imputation with HRC achieved the highest imputation accuracy and allele frequency concordance for the entire minor allele frequency spectrum. Consistent with previous reports, the largest size of the HRC panel among the comparisons is one of the key factors leading to its higher performance [24, 25]. The ethnic composition of the HRC panel could also affect the resulting imputation quality. The samples in the study cohort aligned well with the European population, therefore having a large set of individuals of European ancestry that added to an additional diverse set of 1000G in the HRC panel could also have caused the increased imputation yield [24]. Besides the discussed reasons, there are some minor factors, such as distribution and density of markers in the panel, sequencing coverage and age of the sequenced cohort, leading to a shift in allele frequencies, which could also affect final imputation performance [24, 25].

The overall quality of the imputation achieved with HRC for common variation was consistent with that observed for other ethnicities [24–27]. Imputation of low frequency variants expectedly is less accurate, similarly to many other studies and can benefit from the yet to be created population-specific Russian reference panel [27–31].

The availability of genetic data of Russian descent that would be useful for the creation of the imputation panel is significantly limited. Several sequencing projects, such as Estonian Biocentre Human Genome Diversity Panel (EGDP) [20], Simons Genome Diversity Project (SGDP) [21], and Human Genome Diversity Project (HGDP) [11], have a limited number of samples representing several subpopulations from Russia available for merging in the combined imputation platform (N = 231). However, technical differences in sequencing and data processing between these projects could lead to multiple challenges in homogenizing the whole genome sequencing data across the dataset.

Usage of the UK biobank summary statistics demonstrated that PGS for Russian-descent individuals could be estimated with the quality slightly less than that of the UK Biobank participants. However, due to the reasonably close haplotype structure, the transferability of the UK biobank GWAS results to the northwestern Russian population is more appropriate than to more distant populations (e.g. East Asian) or yet to be studied more eastern Russian ethnicities [1].

There are some potential indirect effects that could influence the PGS estimates for the study cohort. The older age of the Russian cohort to a limited extent might interfere with the estimate of phenotypic variance explained by PGS—the elderly population will generally have ’healthier’ PGS (lower compared to random population snapshot in Russia).

Consistent with the certain lack of transferability of the GWAS results observed previously between populations, the phenotypic variance explained by PGS depends on the allele frequency concordance between UKBB and the study cohort for variants used in the PGS model [4, 32–34].

After careful quality filtration, we found a set of variants with allele frequencies strongly differing between the northwestern Russian and British (UKBB) cohorts, demonstrating a potential source of a decrease in the quality of PGS or GWAS studies compared to the UKBB.

In conclusion, it is important to note that we considered only individuals from the Saint-Petersburg area, yet there are more than 100 ethnic populations in Russia, some of which belong to non-European continental ancestries; therefore, an inclusive approach to polygenic trait studies is especially of great demand in Russia.

## Supporting information

S1 Appendix Additional experiments, technical details, figures, and tables. (PDF)

S1 File (GZ)

S2 File (GZ)
