# Supplementary material for: Genotype imputation and polygenic score estimation in northwestern Russian population
Source: PLoS One. 2022 Jun 28;17(6):e0269434. doi: 10.1371/journal.pone.0269434 (PMC9239469; doi:10.1371/journal.pone.0269434)
Supplement: S1 Appendix — (PDF) [file pone.0269434.s001.pdf]

Genotype imputation and polygenic score estimation in northwestern Russian population

S1 Appendix

Nikita Kolosov[1-3], Valeriia Rezapova[1-3], Oxana Rotar[1,4], Alexander Loboda[1-3,5], Olga Freylikhman[1], Olesya Melnik[1], Alexey Sergushichev[2], Christine Stevens[3], Trudy Voortman[4,6], Anna Kostareva[1], Alexandra Konradi[1,2], Mark J. Daly[3,5,7], Mykyta Artomov[1-3,5,7,*]

[1] Almazov National Medical Research Centre, Saint-Petersburg, Russia
[2] ITMO University, Saint-Petersburg, Russia
[3] Broad Institute, Cambridge, USA
[4] Erasmus MC, University Medical Center, Rotterdam, the Netherlands
[5] Analytic and Translational Genetics Unit, Massachusetts General Hospital, Boston, USA
[6] Division of Human Nutrition & Health, Wageningen University, the Netherlands
[7] Institute for Molecular Medicine Finland (FIMM), Helsinki, Finland

* Corresponding author
E-mail: artomov@broadinstitute.org

The authors declare no conflict of interests.

Table of contents:

Technical Pipeline 3

Phenotypic summary of participants 3

Study Data Processing 3

Reference Panel Processing 4

Pre-imputation Study Data Processing 5

Dataset Quality Filtering for Polygenic Score Estimation 5

Extended Results 6

Genotype Imputation Accuracy 6

Polygenic Score Calculations 12

Population structure 14

Allele frequency discordance of PGS variants 15

Allele frequencies validity check 16

References 40

Technical Pipeline

Phenotypic summary of participants

All participants (n=239) were old age Russians from Saint-Petersburg, Russia. Clinical information was obtained by physician specialists during the ambulatory patient visit. All participants provided written informed consent [1]. Here we provide brief characteristics of the analyzed cohort with key phenotypic parameters (**S1 Table**).

S1 Table. Characteristics of the Participants

Characteristic (N=239)	N (%)	Mean (sd)	Median	Range
Age — yr	-	70.3 (2.9)	70	65-89
Sex (%)				
Male	71 (29.7%)	-	-	-
Female	168 (70.3%)	-	-	-
Height — cm	-	163.1 (8.5)	163	140-192
Weight — kg	-	73.7 (15.4)	72	35-124
BMI	-	27.7 (5.2)	27.6	15.6-45
Waist circumference — cm	-	93.3 (14.5)	93	50-150
Hip circumference — cm	-	105.5 (13.3)	106	37-150
DBP — mm hg	-	84.7 (12.5)	83	52-120
SBP — mm hg	-	150.1 (25)	150	95-242
Glucose — mmol/L	-	5.8 (1.6)	5.4	3.6-18.7
TC — mmol/L	-	5.9 (1.4)	5.9	1.0-10.5
TG — mmol/L	-	1.4 (0.9)	1.3	0.4-9.2
HDL — mmol/L	-	1.4 (0.4)	1.36	0.64-3.01

Study Data Processing

The initial data set included 247 individuals and 633,193 variants. Genotyping data was preprocessed to match the data formatting requirements for BEAGLE[2]. We followed the following recommendations for target data processing: https://dx.doi.org/10.17504/protocols.io.xbgfijw.

We got rid of sex chromosomes and ambiguous contigs. For each SNP with missing rsID we generated the new one from chromosome, position and both alleles (PLINK2 [3], *--set-missing-var-ids @:#\$r:\$a*), all additional rsID prefixes (e.g. GSA, seq etc.) were deleted, all duplicated variants, their first entries, (PLINK2, *--rm-dup force-first*;

PLINK1.9 *--list-duplicate-vars ids-only suppress-first)* and non-biallelic variants were removed (PLINK2, *--max-alleles 2*; *--min-alleles 2*). Also, we filtered out all monomorphic variants, variants with allele count less than 5 and call rate less than 95%. All variants were aligned to human reference genome build 37 (Bcftools [4], *--norm*). 17 individuals with a missing genotype rate more than 0.05 were removed. As a result, we kept 230 individuals and 501,100 variants for further analysis.

Reference Panel Processing

Imputation of study genotypes was performed using several reference panels:

First, we tested 1000 Genomes (Phase 3, Version 5) reference panel. Initially, it included 81,271,745 variants (only autosomes) from 2,504 individuals (ALL) [5]: European (EUR=503), East Asians (EAS=504), Africans (AFR=661), South Asian (SAS=489), and Mixed American (AMR=347). The initial VCF file of an already phased 1000G reference panel (GRCh37) was downloaded from http://ftp.1000genomes.ebi.ac.uk/vol1/ftp/release/20130502/. All variants were processed the same way as the study data. Every procedure was performed for each chromosome separately. As a result, we kept 37,522,002 variants for further analysis.

Second, we tested The Human Genome Diversity Project (HGDP) [6] reference panel downloaded from ftp://ngs.sanger.ac.uk/production/hgdp/hgdp_wgs.20190516/: Africans (n=104), Mixed American (n=61), East Asians (n=223), Central South Asians (n=197), European (n=155), Middle East (n=161), Oceania (n=28). It underwent the same filtering procedures as 1000G. However, in contrast to the 1000G panel, the HGDP panel had missing genotypes, therefore, we performed an additional imputation step, without a reference, to estimate the missing genotypes using Beagle 5.2 (*burnin*=3, *iterations*=4, *ref*=None). Also, current HGDP data was released in NCBI build 38 (UCSC hg38), study data and other panels was annotated in NCBI build 37 (UCSC hg19). We converted all coordinates in the HGDP data to NCBI build 37 to bring all genotype data to the same reference. We used Picard's liftOver tools v.2.23.3 (https://github.com/broadinstitute/picard). The chain file was downloaded from the ensemble server (ftp://ftp.ensembl.org/pub/assembly_mapping/homo_sapiens/GRCh38_to_GRCh37.chain.gz). Approximately 5.3% of variants were rejected during genome conversion. As a result, we kept 26,678,803 variants for further analysis.

Finally, we tested the Haplotype Reference Consortium (HRC) [7] reference panel. Initially, it included 39,131,578 SNPs from 27,165 individuals. The HRC Dataset is a combination of genetic data from 20 unique studies, each with their own data sharing policies and restrictions. Thus, we are not allowed to share the dataset. However, there is a possibility to apply for access from EGA: https://www.ebi.ac.uk/ega/studies/EGAS00001001710. The dataset was processed the

same way as 1000G and HGDP panels. As a result, we kept 37,620,211 variants for further analysis.

Each reference panel before the imputation was converted from *vcf* to *bref3* file format using *bref* v.3 tool from Beagle web page (https://faculty.washington.edu/browning/beagle/bref3.18May20.d20.jar). *bref3* is the recommended file format for *ref* parameter in Beagle 5.1. It gives the fastest computational time.

Pre-imputation Study Data Processing

Study data was pre-phased, strand-checked, all missing genotypes were imputed and, finally, split into 22 chromosomes before the imputation. Most of the procedures were performed using Bcftools [4], tools provided with Beagle (conform-gt.jar; https://faculty.washington.edu/browning/conform-gt.html) or Beagle itself (beagle.jar; https://faculty.washington.edu/browning/beagle/beagle.18May20.d20.jar).

Additionally, for masking experiments, we randomly sampled 9% of variants from each chromosome and put them aside as a separate dataset. The resulting truncated dataset was used for imputation. All studied variants (n=47,209) had non-zero MAF in the northwestern Russian population and were present in all used reference panels.

Further, the final dataset was used for genotype imputation with HRC, 1000G and HGDP reference panels. For all performed imputations we used the same parameters: *burnin*=6, *iterations*=12, *imp-segment*=6, *ne*=1000000. Each chromosome was imputed separately to speed up the process.

Dataset Quality Filtering for Polygenic Score Estimation

Initially, we filtered out only well-imputed variants (Dosage-R^2 >= 0.8) and combined them with genotyped variants. Further, quality control was performed using PLINK 1.9 with the following parameters *--maf* 0.01 *--hwe* 1e-6 *--geno* 0.01 *--mind* 0.01. As a result, 7,156,489 variants were kept.

SNPs with a mismatch in target and reference data alleles were resolved in R with *tidyr*, *dplyr*, and *data.table* libraries and all duplicated SNPs in target data were deleted. We performed LD-pruning using PLINK 1.9 with settings: *--indep-pairwise* 200 50 0.25 to remove highly correlated SNPs. We performed PCA using pruned data and the first 10 PCs were used to account for ancestral variance. Further steps - clumping and polygenic risk estimate were conducted in concordance with previously published protocols [8].

Extended Results

Genotype Imputation Accuracy

The imputation accuracy of the panels was measured using the “masking” approach. There is no any convention about what is the right percentage of variants that need to be sampled to confidently represent the population as a whole. We decided that 9% of variants sampled from each chromosome would be enough to estimate imputation accuracy and at the same time would not significantly affect the final imputation performance due to downsampling of variants used for imputation. Thus, before conducting the imputation procedure, we randomly sampled 9% of variants (chromosome-wise) from study data and put them aside until the completion of the imputation pipeline. All studied variants (n=47,209) had non-zero MAF in the northwestern Russian population and were present in all used reference panels. Afterwards, we compared genotypes for these variants in genotyped and imputed datasets to calculate imputation accuracy scores and concordance of produced allele frequencies.

1. *Accuracy Scores Comparison*

We tested three commonly used imputation accuracy measures - concordance rate (CR), squared Pearson correlation and imputation quality score (IQS) - to justify our choice of accuracy measure. All listed accuracy measures make use of the true masked genotypes, in contrast to DR2, thus making it possible to comprehensively evaluate imputation reliability in absence of whole-genome sequencing data for the Russian cohort.

We calculated these scores for all three reference panels (HRC, 1000G, HGDP) to illustrate the reproducibility of observed trends and biases regardless of the selected imputation reference panel (**S1-3 Fig**).

Our results provide further evidence that concordance rate, in comparison with IQS and other studied scores, inflates accuracy estimates particularly for low frequency variants (**S4 Fig**). Besides, CR estimates across different minor allele frequency groups were almost the same for all studied reference panels, thus not displaying actual difference in imputation performance. CR showed a significant, but moderate, correlation with IQS for all three panels (r=0.46-0.58, p-value<$1*10^{-6}$).

Squared Pearson correlation was immeasurable for variants with uniform dosages (MAF=0), thus, making it difficult to calculate reliable accuracy estimates for some fraction of rare variants. More specifically, some masked variants were imputed with uniform dosage dosages, thus having zero MAFs. As a result, there was zero variation in response variable, thus making correlation coefficient between masked and imputed

dosages immeasurable. Also, when allele frequencies were near 0.5 value, square correlation led to the opposite encoding, reversing minor allele. However, there were few of such variants. The squared correlation coefficient was highly correlated with the IQS (r=0.90-0.96, p-value<1*10^{-6}).

DR2 measure, derived from Beagle output, was also significantly correlated with IQS (r=0.87-93, p-value<1*10^{-6}). Differences between them occurred mostly for rare variants (**S1B, S2B, S3B Fig**).

Observed results fit well in the previous reports about other populations [9,10]. Thus, it can be assumed that these accuracy measures - IQS, concordance rate, squared Pearson correlation - can be used regardless of the choice of the reference panel or the studied population.

Altogether, IQS showed the most credible results for imputation quality evaluation for the masked genotyped variants. Therefore, we used it for further comparison of imputation panels in absence of the whole genome sequencing data for Russian-descent individuals.

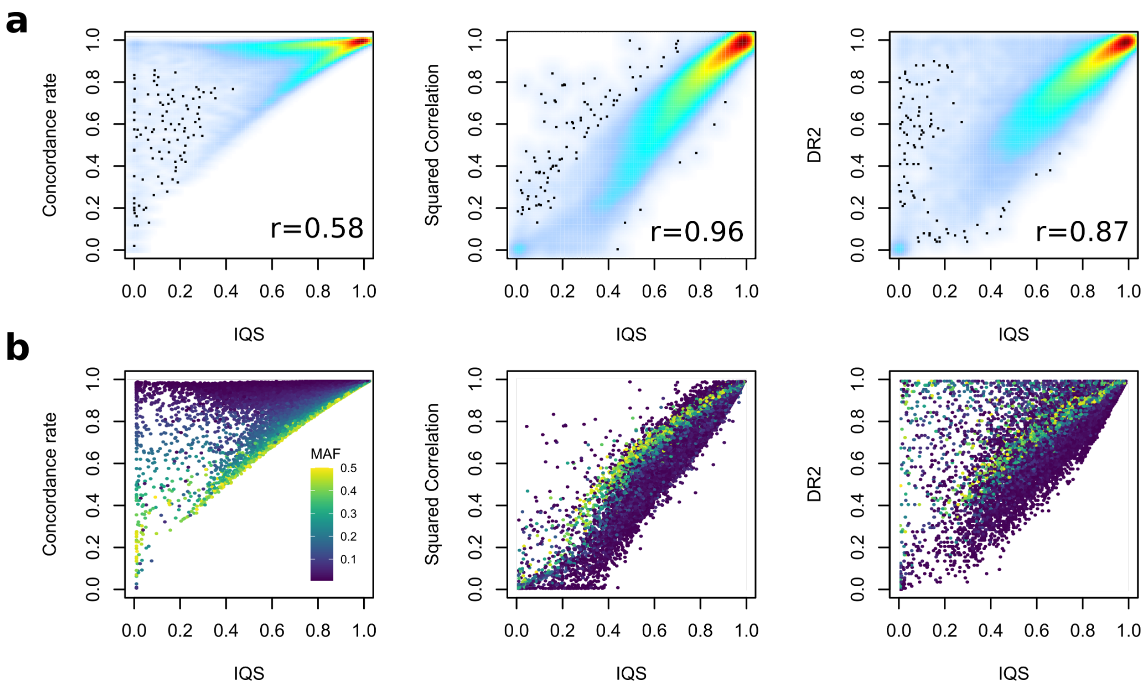

S1 Fig. Correlation of imputation accuracy measures for masked variants, imputed by the Haplotype Reference Consortium reference panel. (a) Smoothed color density representation of a scatter plot (blue - low density, red - high density). **(b)** Scatter plots with color representing MAF for each variant (purple - low MAF, yellow - high MAF).

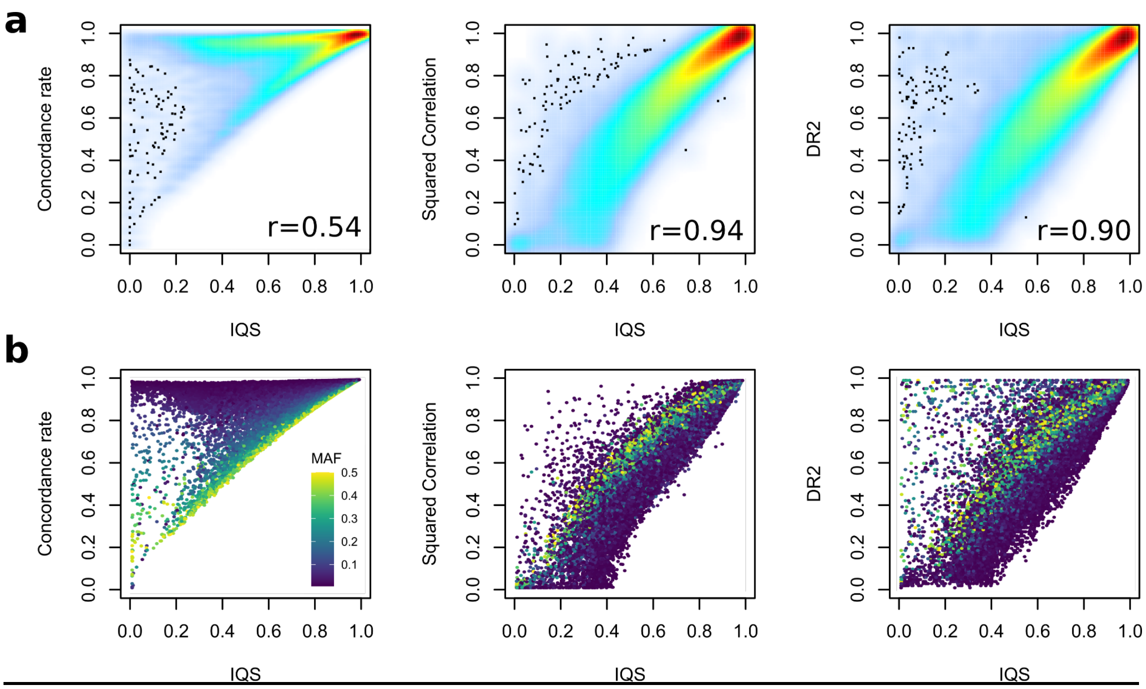

S2 Fig. Correlation of imputation accuracy measures for masked variants, imputed by the 1000 Genomes reference panel. **(a)** Smoothed color density representation of a scatter plot (blue - low density, red - high density). **(b)** Scatter plots with color representing MAF for each variant (purple - low MAF, yellow - high MAF).

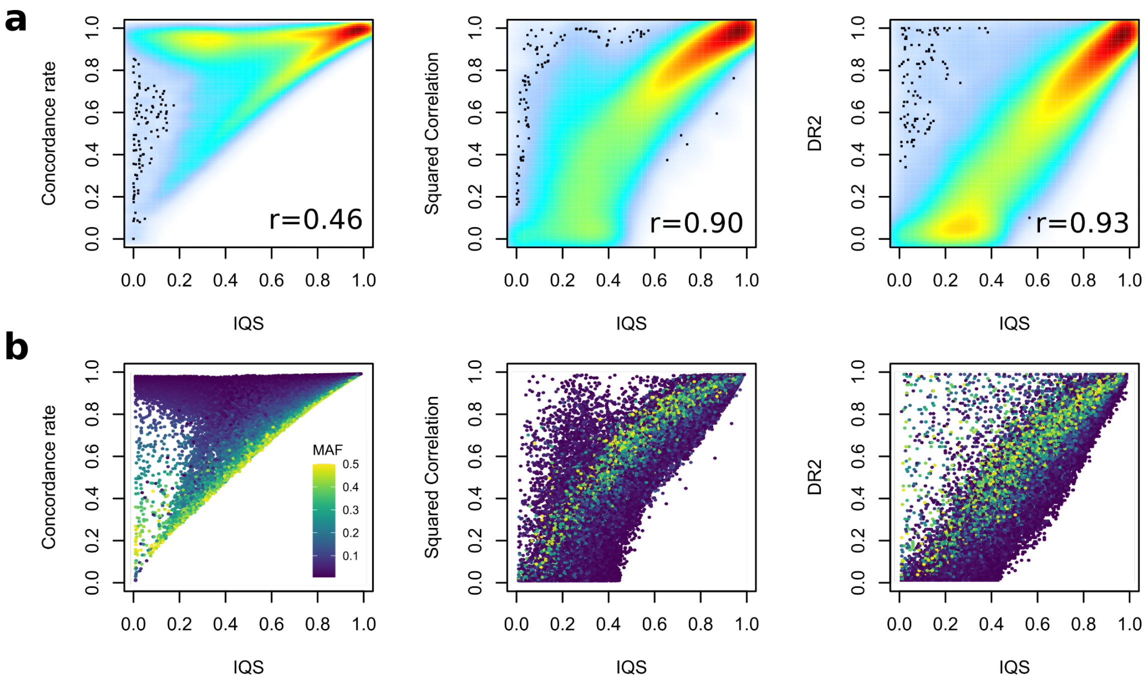

S3 Fig. Correlation of imputation accuracy measures for masked variants, imputed by the Human Genome Diversity Project reference panel. **(a)** Smoothed

color density representation of a scatter plot (blue - low density, red - high density). **(b)** Scatter plots with color representing MAF for each variant (purple - low MAF, yellow - high MAF).

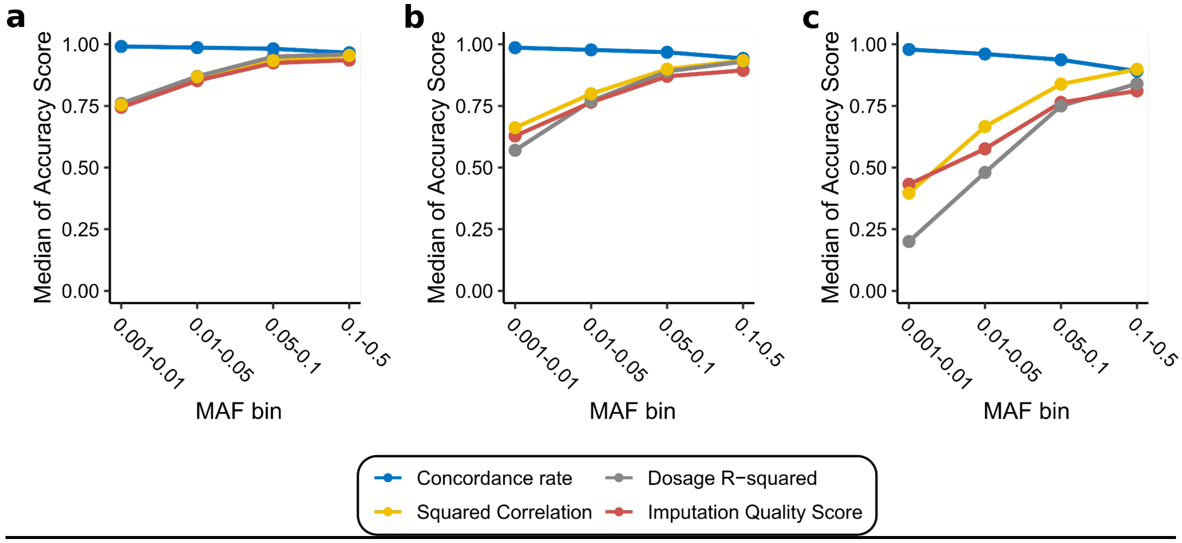

S4 Fig. Comparison of imputation accuracy scores across different minor allele frequency (MAF) groups. Imputation reference panel: **(a)** HRC. **(b)** 1000G. **(c)** HGDP. Blue - Concordance rate, Gray - Dosage R-squared, Yellow - Squared Correlation, Red - Imputation Quality Score.

2. *Evaluation of Imputation Accuracy*

We estimated the relationship between IQS and DR2 for the masked variants to determine the optimal DR2 threshold that minimizes the introduction of variants that are unlikely to be found in the Russian population.

By increasing the DR2 threshold we significantly reduced the resulting imputation yield. For example, using DR2>= 0.8 we kept only 22% of all imputed variants for HRC panel, 21% for 1000G panel and 14% for HGDP panel (**S5A Fig**). However, applying such a strong threshold, we increased expected median imputation accuracy. Threshold DR2 >= 0.8 ensures that the expected IQS to be equal or greater 0.8 for masked variants for all three panels (**S5B Fig**). As well, DR2>=0.8 is one of the commonly used thresholds for defining confidently “well-imputed” variants [11–13].

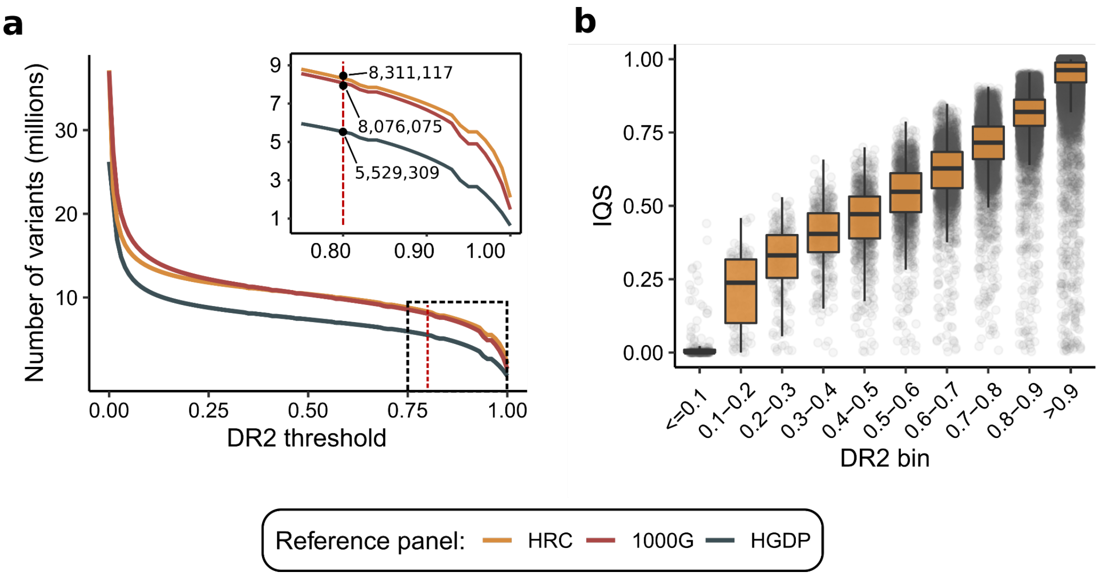

Supplementary Fig S5. Number of variants for different DR2 thresholds and Median IQS values across different DR2 bins. (a) The estimated number of well-imputed variants for different DR2 thresholds. Dashed red line - DR2 = 0.8 **(b)** IQS as a function of DR2 (HRC panel). DR2 - Dosage R-squared, IQS - imputation quality score. HRC - Haplotype Reference Consortium, 1000G - 1000 Genomes project, HGDP - Human Genome Diversity Project.

Supplementary Table S2. Comparison of Median IQS values for different minor allele frequency (MAF) groups between imputation reference panels.

	MAF			
**Panel**	**0.001-0.01**	**0.01-0.05**	**0.05-0.1**	**0.1-0.5**
HRC	0.744	0.852	0.924	0.936
1000G	0.628	0.764	0.869	0.894
HGDP	0.432	0.575	0.764	0.810

3. Allele Frequency Concordance

In addition to accuracy measures, we calculated concordance of allele frequencies between imputed and masked variants for all three panels. Though concordance of allele frequencies is not an accuracy score, it allows us to measure imputation reliability in terms of accordance of imputed frequencies to the observed ones in a more explicit way.

Variants imputed by the HRC reference panel, in comparison with HGDP and 1000G panels, showed the most concordant allele frequencies in terms of both the lowest

number of concordant variants (n=607, ~1%) and the lowest Mean Absolute Error (MAE) between imputed and observed frequencies (MAE=0.007). For more details see **S3 Table, S6 Fig**.

S3 Table. Comparison of imputation reference panels based on several allele frequency concordance metrics.

Panel	Concordant SNPs	Discordant SNPs	Disc./Total (%)	MAE
HRC	46601	607	1.28%	0.007
1000G	45529	1679	3.55%	0.011
HGDP	38712	8494	17.99%	0.021

Altogether, we determined that HRC is an optimal imputation reference panel for imputing genotypes from a Russian cohort based on imputation accuracy and allele frequency concordance measures between masked and imputed variants (**S2** and **S3 Table**).

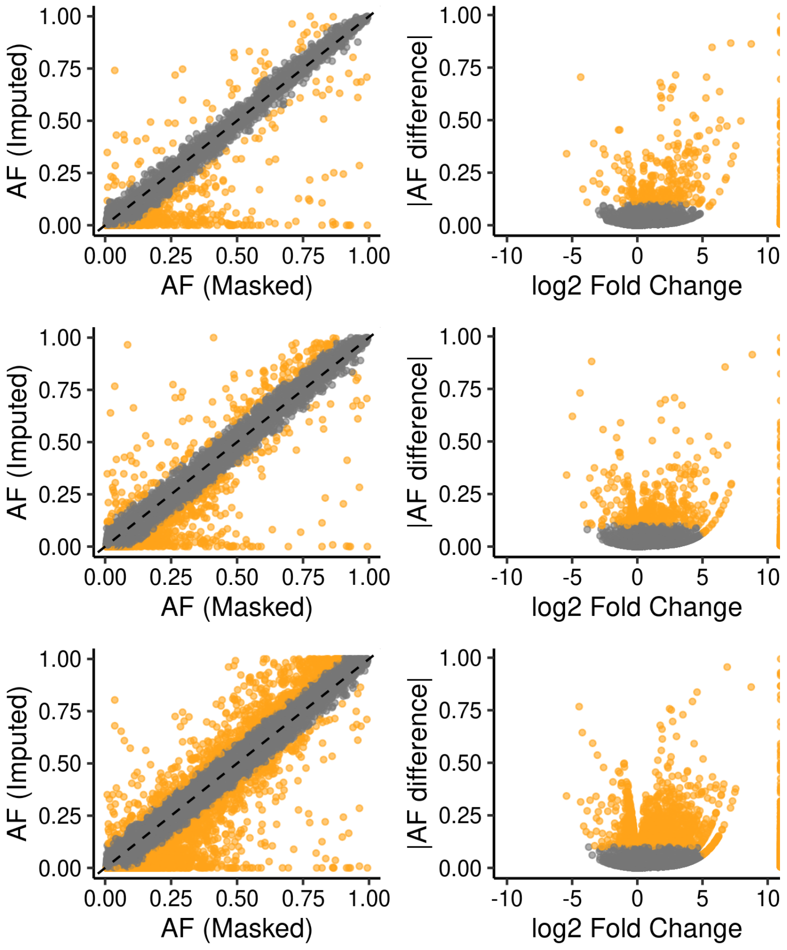

S6 Fig. Concordance of allele frequencies between imputed and masked variants. Imputation reference panel: **(a)** HRC. **(b)** 1000G. **(c)** HGDP. AF - alternative allele frequency, Gray - concordant variants, Orange - discordant variants, Dashed line - ideal concordance model, when imputed allele frequencies totally match masked allele frequencies.

Polygenic Score Calculations

GWAS summary statistics for 11 phenotypes (body mass index 21001_irnt, weight 21002_irnt, height 50_irnt, waist 48_irnt, and hip 49_irnt circumferences, diastolic 4079_irnt and systolic 4080_irnt blood pressure, triglyceride 30870_raw, cholesterol 30690_raw, and glucose 30740_raw levels, high-density lipoprotein 30760_raw level) was downloaded from UKBB spreadsheet (https://docs.google.com/spreadsheets/d/1kvPoupSzsSFBNSztMzl04xMoSC3Kcx3CrjVf4yBmESU/edit#gid=178908679).

First, we kept only variants that were present in both UKBB summary statistics and the Russian cohort. Further, variants were clumped using PLINK 1.9 with the following

flags *--clump-p1 0.01 --clump-p2 1 --clump-r2 0.9 --clump-kb 250*. We computed a series of polygenic scores using variants passing different thresholds of p-values in summary statistics ($5x10^{-8}$, $1x10^{-6}$, $1x10^{-4}$, $1x10^{-3,}$ and $1x10^{-2}$) for further parameter optimization steps.

We followed the protocol described in Martin et al to construct models for evaluation of concordance of the polygenic score with actual clinical data. Full linear model was given as: phenotype ~ PGS + age + age^2 + sex + sex*age + sex*age^2 + PC(1-10). The nested model included all the same covariates except PGS. R^2 attributed to PGS was estimated as the difference between R^2 of the full and the nested models. We used the optimal p-value threshold in the summary statistics delivering the largest R^2 for PGS (**S7** and **S8 Fig**).

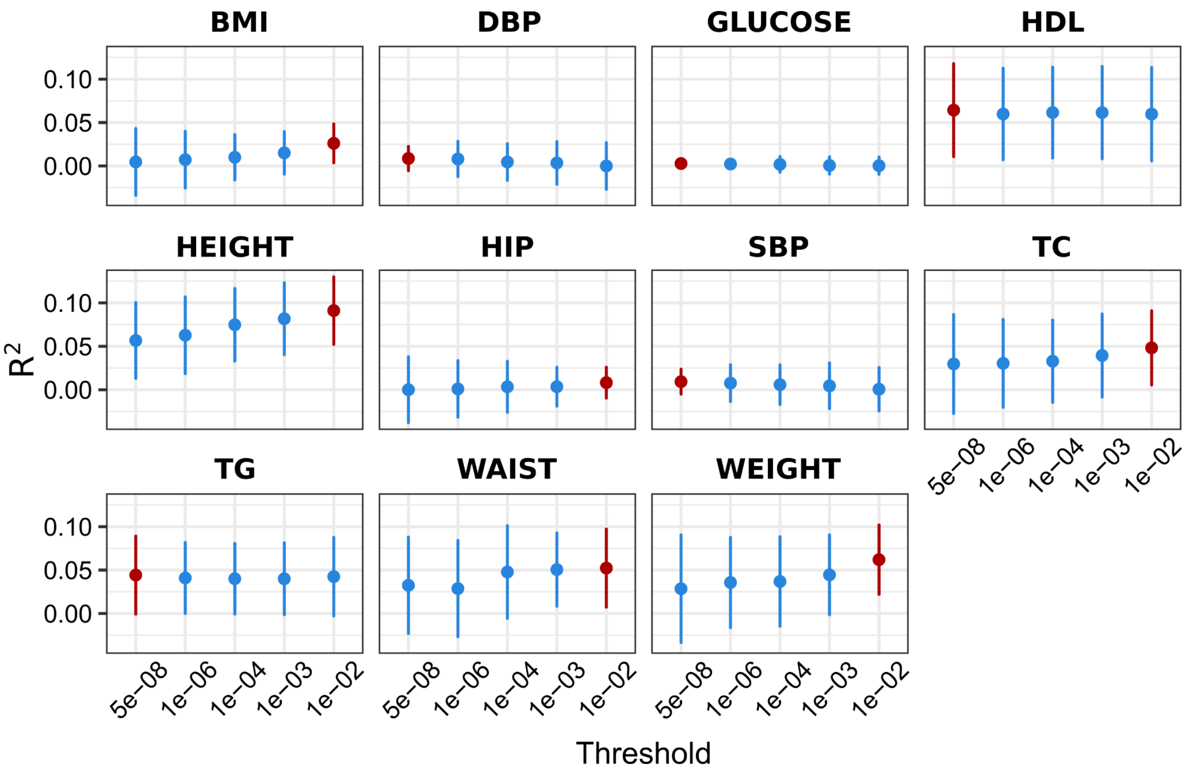

S7 Fig. Optimal P-value threshold selection for imputed genotyping data. 95% confidence intervals were calculated by bootstrapping (1000 iterations). Red - optimal p-value threshold.

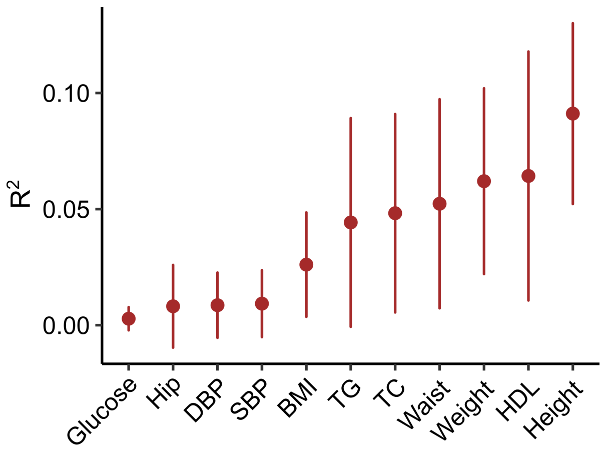

S8 Fig. Phenotypic variance explained by polygenic scores

Population structure

We showed, based on PCA analysis and Wright's Fixation index, that northwestern Russian individuals in general are genetically similar to the European population.

1. *Principal component analysis (PCA)*

We performed PCA to compare haplotype structure between Russian-descent individuals and other populations from a 1000G data set (**Fig 2C)**. The 1000G reference panel consists of 5 continental populations: EUR, AMR, AFR, EAS, SAS. We took the already phased study data and 1000G reference panel and extracted only variants presented in both datasets (Bcftools, *--isec, -n*=*2*). Further, we performed LD pruning along with SNP quality filtering (PLINK2, *--maf* 0.01 *--hwe* 0.0001 *--indep-pairwise* 50 5 0.5). Any SNP with strand bias wasn't included in the combined data set. After all the procedures, we kept 247,495 variants. The PCA was performed using PLINK2 *--pca* function. The PCA plot was generated using R v.3.6 and the ggplot package (https://github.com/tidyverse/ggplot2). We used the first two principal components to reflect the genotype variability (PC1 - 12.62%; PC2 - 8.34%). Northwestern Russian individuals are co-localized with the European population in 1000 Genomes.

2. *Wright's Fixation index*

Further, we evaluated the genetic differences between Russian population and each population from a 1000G dataset by calculating pairwise Weir and Cockerham's fixation index (F_{st}) [14] (**S9 Fig**). We took only variants with minor allele count more or equal three in all studied populations (n=337,405). F_{st} was computed individually for each autosome using VCFtools [15] and then averaged.

To compare continental cohorts (EUR, AMR, AFR, EAS, SAS) between each other we calculated the mean F_{st} value based on F_{st} values from individual populations within the cohort. Populations of European-ancestry (EUR) showed the lowest mean F_{st} values (F_{st}=0.0056) in comparison with others, thus showing the smallest differentiation from the Russian cohort. South Asians (SAS) was the second most similar cohort (F_{st}=0.0367). For AMR, EAS and AFR populations, F_{st} values were estimated to be 0.0382, 0.1044 and 0.1313, respectively.

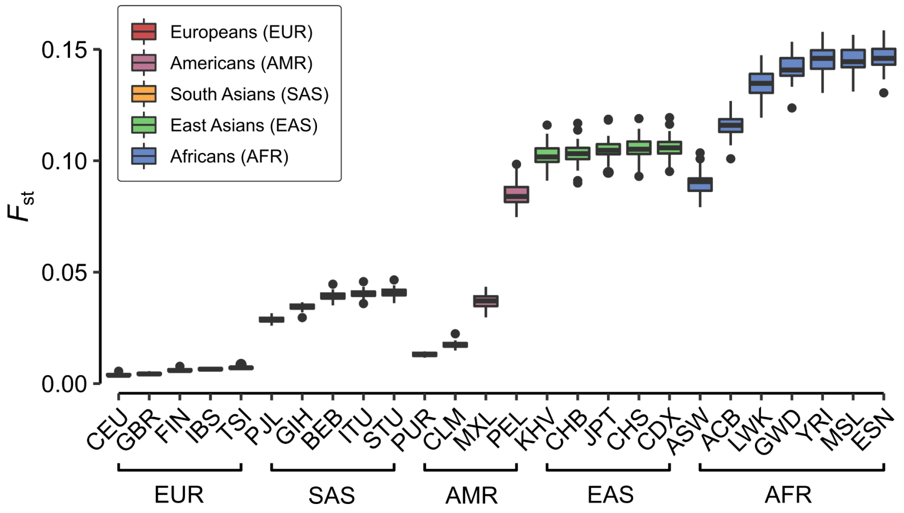

S9 Fig. Distribution of Fixation index (F_{st}) values for each population from 1000G. Each data point corresponds to the weighted mean of F_{st} for each chromosome. Thus each boxplot consists of 22 (number of tested autosomes) data points. The closer the mean index value to zero the closer the tested population is to the Russian cohort based on the whole-genome index value.

Allele frequency discordance of PGS variants

We extracted allele frequencies for variants used in PRS estimation from both UKBB summary statistics and Study cohort. Further, we calculated mean absolute errors (MAE) between these two datasets for each PRS model. MAE in this case stands for the allele frequency discordance measure. Notably, the PRS model for glucose level included considerably less variants than other models and explained almost none of the

phenotypic variance, thus we decided to exclude this model from the analysis (**S10a Fig**). Then, we compared MAE values with the incremental-R^2 achieved by the PRS models to check the linear relationship between the degree of divergence of allele frequencies and phenotypic variance explained by the PRS models. As a result, we get negative correlation (R^2=0.51, *p*-value=0.0119, **S10b Fig**). Thus, the more allele frequencies between study cohort and summary statistics are discordant, the less PRS models are informative.

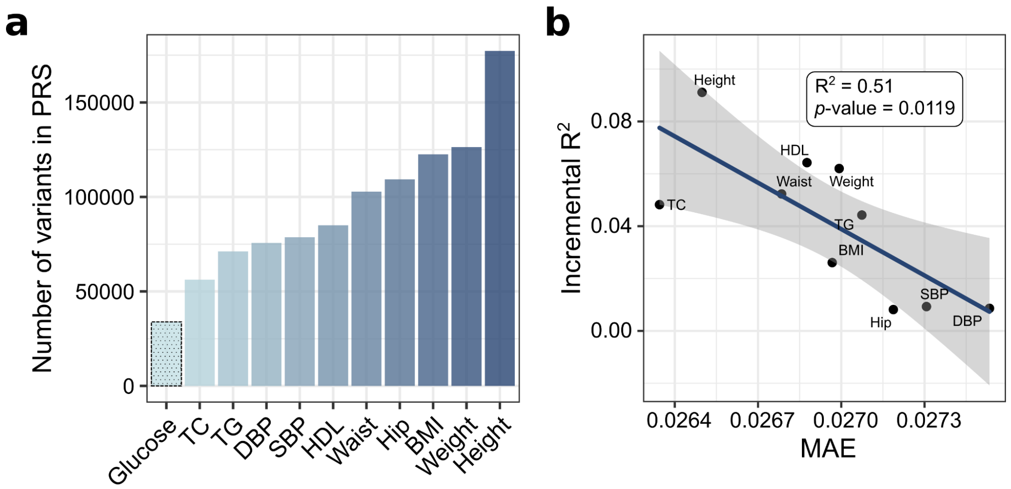

S9 Fig. Allele frequency concordance comparison for variants used in PRS models. (a) Total number of variants in PRS models for 11 phenotypes. (b) Regression analysis between mean absolute errors and R^2-increments.

Allele frequencies validity check

In this section we describe all of the intermediate steps and quality check procedures that we conducted prior to allele frequency concordance analysis. In that manner we sought to eliminate all of the potential technical artifacts that could arise during genotyping procedure or preprocessing steps (**S4 Table**).

We believe that such strict quality check procedures are exceedingly conservative and likely eliminate a lot of true-positive variant calls, however, they allow to minimize the chances of picking up a technical artifact. This is especially important, since we functionally annotate and draw conclusions from these variants.

1. Initial datasets preprocessing

Initially, all of the datasets used in the analysis (1KG [5], HGDP [6], EGDP [16] and the study dataset) were aligned to the same reference genome (NCBI build 37).

Strand-orientation misalignment artifacts were checked using *bcftools +fixref* plugin (https://samtools.github.io/bcftools/howtos/plugin.fixref.html). All of the variants from aforementioned datasets had CHR:POS:REF:ALT variant id format, thus we knew alternative alleles and their frequencies for all of the variants in all of the datasets and we compared only variants with matched REF/ALT alleles.

2. Study data quality control

Initially, study data variants were filtered with GTS (GenTrain score) >= 0.3, Call Rate 0.95, AF != 0. These were standard thresholds before RUS-UKBB allele frequency comparison to ensure that we eliminate potential technical artifacts (**S11a Fig**). Further, we decided to apply more strict quality check thresholds: HWE >= 1e-06, GTS >= 0.5, ExcHet > 0.05, call rate >= 0.97 (**S11b Fig**).

3. UKBB dataset quality control

Next, we hypothesized that some of the technical noise could be due to general heterogeneity of data sources used to acquire variants for the UKBB dataset. Specifically, some of the individuals (n~50k) were genotyped using UK BiLEVE Axiom array, others (n~450k) - UK Biobank Axiom array. Also, some of the variants were imputed using a 10KUK panel, others - using a merged 1000G+HRC panel. Thus, we decided to additionally filter the UKBB dataset: INFO > 0.9, call rate > 0.97, HWE (p-value) > 1e-06, ExcHet > 0.05, AF != 0 (**S11c Fig**). And, further, we get rid of all variants whose alternative allele frequencies were discordant (logFC > 5 or logFC < -5 or AF difference > 0.10) with the 10KUK dataset (**S11d Fig**).

Altogether, all of the variants from UKBB were filtered using standard QC scores, all of the allele frequencies were concordant with 10KUK WGS dataset.

4. Allele frequency confirmation

Finally, we compared the alternative allele frequencies (AFs) in the study dataset with Russian AFs from the external WGS datasets. First, we obtain an EGDP dataset that contains Russian individuals from several geographical regions (26 - Caucasus, 45 - European part of Russia, 102 - Siberia). Using these sets we constructed three allele frequency lists, specifically, for each group and one for the combined set (N=173). Further, we do the same using 25 Russian individuals (Arkhangelsk region) from the HGDP dataset. To ensure we filtered out technical artifacts, we kept for the analysis only variants that had concordant (logFC within +/-5 region; AF difference <= 0.2) allele frequencies at least in one of the comparisons (**S11e** and **S12 Fig**).

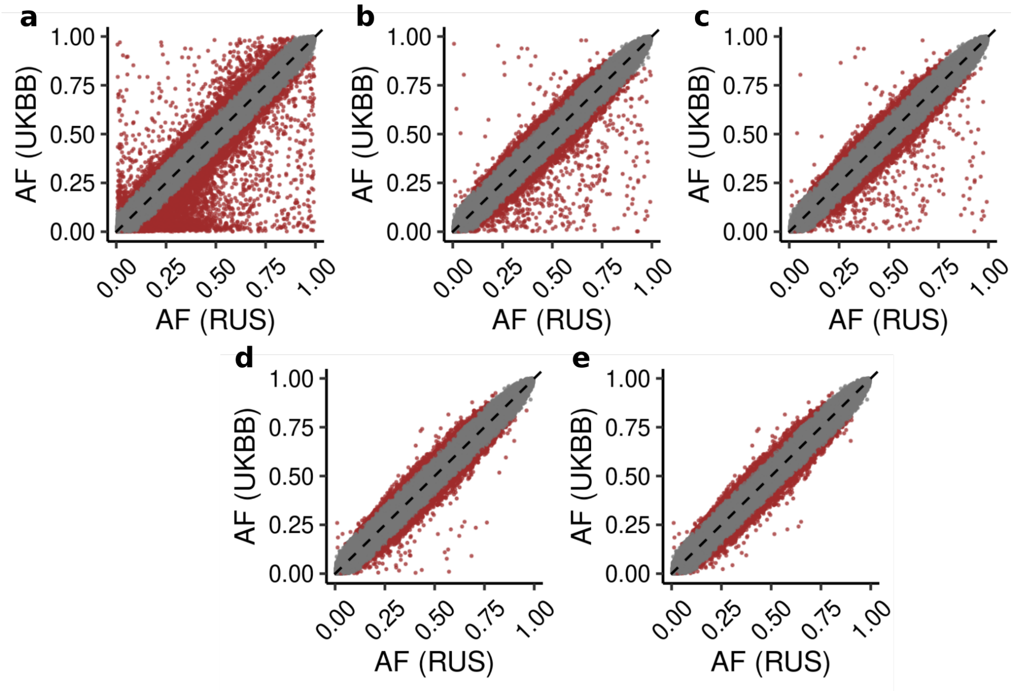

S11 Fig. Concordance of allele frequencies between northwestern Russian (RUS) and Great-Britain populations (UKBB). Red - discordant variants, Gray - concordant variants. Detailed description of each QC-filtering step is given in the text.

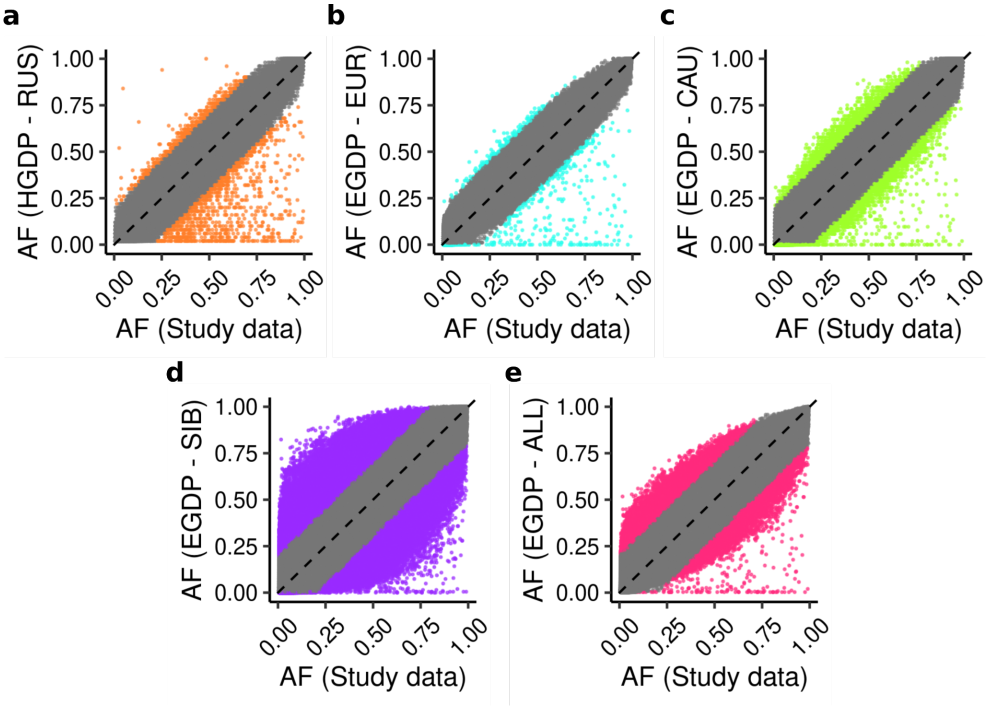

S12 Fig. Alternative allele frequencies comparison between Study data and several WGS datasets: (a) HGDP (Russian subset) = 25; (b) EGDP (European part of Russia) = 45; (c) EGDP (Caucasus) = 26; (d) EGDP (Siberia) = 102; (e) EGDP (All Russian populations combined) = 173. Gray - concordant variant, colored - discordant variant.

S4 Table. Total number of variants and number of discordant variants in study data on different quality control steps

Quality control steps	Total number of variants	Number of disc. variants	Number of disc. Variants (%)
Initial filtering	478,334	7,610	1.56
Increased study data filtering	405,672	3,218	0.79
Increased UKBB SS filtering	397,506	2,958	0.74
Keep only conc. vars between UKBB & 10UK datasets	387,128	2,572	0.66
Keep only conc. vars between study data and HGDP-EGDP datasets	379,751	2,523	0.66

S5 Table. Traits from GWAS catalog significantly associated with the identified deviated variants between RUS-UKBB.

GWAS catalog trait	RSID	P-value	UKBB AF	CEU AF	FREQ AF
vWF levels	rs687289	5E-1443	0.578966	0.555556	0.461538
Mean platelet volume	rs342293	8E-1151	0.712292	0.691919	0.595142
Factor VIII levels	rs687289	7E-778	0.578966	0.555556	0.461538
Factor VIII levels	rs687621	1E-647	0.650877	0.671717	0.753036
Serum alkaline phosphatase levels	rs492602	1E-566	0.476057	0.449495	0.609312
Lymphocyte counts	rs2249742	6E-421	0.580392	0.575758	0.692308
White blood cell count	rs2249742	4E-405	0.580392	0.575758	0.692308
Platelet distribution width	rs342293	3E-395	0.712292	0.691919	0.595142
vWF levels	rs687621	4E-324	0.650877	0.671717	0.753036
Platelet count	rs342293	9E-319	0.712292	0.691919	0.595142
Ankylosing spondylitis	rs7743761	5.00E-304	0.480198	0.489899	0.599594
Psoriasis or type 2 diabetes (trans-disease meta-analysis)(opposite effect)	rs10484554	2.00E-302	0.677902	0.707071	0.558704
Psoriasis	rs10484554	4.00E-214	0.677902	0.707071	0.558704
Cutaneous psoriasis	rs10484554	4.00E-207	0.677902	0.707071	0.558704
Lung function (FEV1/FVC)	rs13141641	4.00E-184	0.739669	0.737374	0.621951
Blood protein levels	rs7522061	7.00E-182	0.354328	0.318182	0.457317
Venous thromboembolism	rs687289	1.00E-174	0.578966	0.555556	0.461538
Tissue factor levels	rs492602	1.00E-142	0.476057	0.449495	0.609312
Tumor biomarkers	rs17271883	5.00E-132	0.83241	0.883838	0.717213
Coagulation factor levels	rs687289	1.00E-128	0.578966	0.555556	0.461538
Peak expiratory flow	rs13141641	7.00E-126	0.739669	0.737374	0.621951
Blood protein levels	rs778809	1.00E-118	0.632169	0.59596	0.491837
Blood protein levels	rs7528684	1.00E-112	0.522883	0.565657	0.629098
Activated partial thromboplastin time	rs687621	9.00E-100	0.650877	0.671717	0.753036
Myeloid white cell count	rs2249742	5.00E-71	0.580392	0.575758	0.692308
Activated partial thromboplastin time	rs687289	9.00E-68	0.578966	0.555556	0.461538

Chronic hepatitis B infection	rs7453920	1.00E-60	0.375118	0.39899	0.483607
Mean corpuscular volume	rs725518	1.00E-59	0.400942	0.383838	0.274793
IgG N-glycosylation phenotypes (multivariate analysis)	rs11847263	1.00E-58	0.386088	0.39899	0.493902
Serum metabolite levels	rs2242086	1.00E-55	0.266754	0.313131	0.165992
Amyloid A serum levels	rs4638289	3.00E-53	0.570429	0.5	0.680162
Venous thromboembolism	rs687621	2.00E-52	0.650877	0.671717	0.753036
Waist-to-hip ratio adjusted for BMI	rs9991328	1.00E-51	0.230671	0.30303	0.346154
Mean corpuscular hemoglobin	rs725518	2.00E-51	0.400942	0.383838	0.274793
Receptor for advanced glycosylation end products levels	rs204993	1.00E-48	0.504532	0.59596	0.623984
FEV1	rs13141641	4.00E-48	0.739669	0.737374	0.621951
Idiopathic membranous nephropathy	rs2233956	6.00E-48	0.56275	0.535354	0.698381
Blood metabolite levels	rs7570971	8.00E-45	0.283609	0.247475	0.392713
Cerebrospinal fluid levels of Alzheimer's disease-related proteins	rs573521	2.00E-44	0.318894	0.292929	0.453441
Idiopathic membranous nephropathy	rs3130564	1.00E-42	0.613219	0.671717	0.733607
Mean platelet volume	rs397969	9.00E-42	0.396805	0.439394	0.538618
Human milk oligosaccharide concentration (lacto-N-fucopentaose I)	rs492602	4.00E-41	0.476057	0.449495	0.609312
Chronic obstructive pulmonary disease	rs13141641	9.00E-41	0.739669	0.737374	0.621951
Blood protein levels	rs687289	1.00E-40	0.578966	0.555556	0.461538
Psoriasis	rs13437088	3.00E-40	0.228459	0.29798	0.330612
Blood metabolite ratios	rs4150581	2.00E-37	0.60898	0.606061	0.508097
Hepatitis B	rs7453920	5.00E-37	0.375118	0.39899	0.483607
Urinary metabolites (H-NMR features)	rs2287921	3.00E-36	0.222333	0.212121	0.109312
Height	rs6457374	8.00E-35	0.474429	0.469697	0.364372
Blood protein levels	rs6457374	2.00E-34	0.474429	0.469697	0.364372
Diastolic blood pressure (cigarette smoking interaction)	rs1378942	8.00E-33	0.180921	0.136364	0.283673
Inflammatory bowel disease	rs7134599	9.00E-32	0.290886	0.267677	0.396341
Keratinocyte cancer (MTAG)	rs2153271	5.00E-31	0.684628	0.722222	0.802846

Blood metabolite levels	rs2403254	1.00E-30	0.29284	0.292929	0.188259
Monocyte count	rs1967309	1.00E-30	0.354484	0.368687	0.45625
Diastolic blood pressure (cigarette smoking interaction)	rs6495122	5.00E-30	0.300596	0.5	0.495935
Alanine aminotransferase levels	rs687621	9.00E-30	0.650877	0.671717	0.753036
Diastolic blood pressure	rs6495122	3.00E-29	0.300596	0.5	0.495935
Protein quantitative trait loci	rs3761959	3.00E-29	0.322011	0.318182	0.219512
Heel bone mineral density	rs2761884	5.00E-29	0.247889	0.181818	0.369919
Height	rs2256183	8.00E-29	0.316218	0.378788	0.427126
Hip circumference adjusted for BMI	rs9266043	8.00E-29	0.368435	0.373737	0.477459
Mean arterial pressure	rs11072508	8.00E-29	0.384867	0.323232	0.267347
Waist-to-hip ratio adjusted for BMI	rs12936587	8.00E-29	0.43639	0.409091	0.554878
Matrix metalloproteinase levels	rs11225434	9.00E-29	0.528752	0.60101	0.425101
Systolic blood pressure (cigarette smoking interaction)	rs1378942	1.00E-28	0.180921	0.136364	0.283673
CD4:CD8 lymphocyte ratio	rs2524054	2.00E-28	0.24134	0.242424	0.637652
Erythrocyte sedimentation rate	rs12034383	2.00E-28	0.232127	0.232323	0.332645
Heel bone mineral density	rs12477810	2.00E-28	0.609873	0.570707	0.493827
Waist-hip ratio	rs9991328	5.00E-28	0.230671	0.30303	0.346154
Mean corpuscular hemoglobin	rs4133213	2.00E-27	0.565055	0.565657	0.443089
Mean reticulocyte volume	rs725518	2.00E-27	0.400942	0.383838	0.274793
Blood protein levels	rs516246	4.00E-27	0.195157	0.212121	0.295918
Diastolic blood pressure	rs1378942	3.00E-26	0.180921	0.136364	0.283673
Low density lipoprotein cholesterol levels	rs7741091	3.00E-26	0.419736	0.39899	0.548583
Systolic blood pressure	rs699	6.00E-26	0.551494	0.676768	0.691057
Monocyte count	rs687289	8.00E-26	0.578966	0.555556	0.461538
Blood metabolite levels	rs8051149	9.00E-26	0.000326694	0.00505051	0.0182186
Retinal vascular caliber	rs2287921	2.00E-25	0.222333	0.212121	0.109312
Neutrophil count	rs2249742	6.00E-25	0.580392	0.575758	0.692308
Estimated glomerular filtration rate	rs34468415	1.00E-24	0.355492	0.348485	0.516194
IgG glycosylation patterns	rs11847263	2.00E-24	0.386088	0.39899	0.493902
Mean spheric corpuscular volume	rs725518	2.00E-24	0.400942	0.383838	0.274793

Hip circumference adjusted for BMI	rs2261033	3.00E-24	0.175818	0.181818	0.298781
Cardiovascular disease	rs11072508	5.00E-24	0.384867	0.323232	0.267347
Alzheimer's disease (late onset)	rs6656401	6.00E-24	0.587893	0.575758	0.693089
Breast cancer	rs2046210	6.00E-24	0.190511	0.166667	0.340081
Waist-hip ratio	rs12936587	6.00E-24	0.43639	0.409091	0.554878
Parkinson's disease	rs6430538	8.00E-24	0.46177	0.484848	0.565041
Hip circumference adjusted for BMI	rs630379	2.00E-23	0.0906701	0.0808081	0.210526
Human milk oligosaccharide concentration (2'-fucosyllactose)	rs2287921	2.00E-23	0.222333	0.212121	0.109312
Human milk oligosaccharide concentration (lacto-N-fucopentaose II)	rs516246	4.00E-23	0.195157	0.212121	0.295918
Systolic blood pressure (cigarette smoking interaction)	rs6495122	4.00E-23	0.300596	0.5	0.495935
Systolic blood pressure	rs1378942	6.00E-23	0.180921	0.136364	0.283673
Prostate cancer	rs12155172	9.00E-23	0.0478095	0.020202	0.157895
IgG glycosylation	rs11847263	1.00E-22	0.386088	0.39899	0.493902
Type 1 diabetes and autoimmune thyroid diseases	rs2251396	1.00E-22	0.416044	0.419192	0.605263
Systemic lupus erythematosus	rs2051549	3.00E-22	0.343306	0.373737	0.46473
Macular thickness	rs9398171	6.00E-22	0.514964	0.5	0.617409
Ganglion cell inner plexiform layer (GCIPL) thickness	rs9398171	8.00E-22	0.514964	0.5	0.617409
Retinal nerve fibre layer thickness or ganglion cell inner plexiform layer thickness (MTAG)	rs9398171	8.00E-22	0.514964	0.5	0.617409
Atrial fibrillation	rs2145587	2.00E-21	0.421225	0.444444	0.530488
Blood protein levels	rs492602	2.00E-21	0.476057	0.449495	0.609312
Follicular lymphoma	rs2647012	2.00E-21	0.414683	0.459596	0.311741
Serum alkaline phosphatase levels	rs34705571	2.00E-21	0.781233	0.777778	0.634146
Alcohol consumption (drinks per week) (MTAG)	rs281379	4.00E-21	0.34576	0.318182	0.447154
Alcohol consumption (drinks per week)	rs281379	5.00E-21	0.34576	0.318182	0.447154
Blood protein levels	rs1061098	5.00E-21	0.155509	0.181818	0.301619
Mean corpuscular hemoglobin	rs2158007	5.00E-21	0.575455	0.530303	0.471074

CD8-positive T-lymphocyte count	rs2524054	6.00E-21	0.24134	0.242424	0.637652
Medication use (agents acting on the renin-angiotensin system)	rs11072508	9.00E-21	0.384867	0.323232	0.267347
Crohn's disease	rs516246	1.00E-20	0.195157	0.212121	0.295918
Metabolic traits	rs2403254	1.00E-20	0.29284	0.292929	0.188259
Total cholesterol levels	rs492602	1.00E-20	0.476057	0.449495	0.609312
Total cholesterol levels	rs516246	1.00E-20	0.195157	0.212121	0.295918
Heel bone mineral density	rs4466675	2.00E-20	0.326265	0.353535	0.432377
Folate pathway vitamin levels	rs602662	3.00E-20	0.294849	0.242424	0.192308
Metabolic traits	rs10799701	3.00E-20	0.157677	0.10101	0.267206
Itch intensity from mosquito bite	rs521977	4.00E-20	0.533993	0.50505	0.421162
PR interval	rs7692808	6.00E-20	0.680172	0.681818	0.55668
Blood metabolite levels	rs9302065	1.00E-19	0.613119	0.60101	0.716599
Hip circumference adjusted for BMI	rs1589163	1.00E-19	0.367304	0.363636	0.487552
Post bronchodilator FEV1/FVC ratio	rs13141641	1.00E-19	0.739669	0.737374	0.621951
Gut microbiota abundance (genus Bifidobacterium id.436)	rs7570971	2.00E-19	0.283609	0.247475	0.392713
Gut microbiota abundance (family Bifidobacteriaceae id.433)	rs7570971	3.00E-19	0.283609	0.247475	0.392713
Gut microbiota abundance (order Bifidobacteriales id.432)	rs7570971	3.00E-19	0.283609	0.247475	0.392713
Cardiovascular disease	rs516246	4.00E-19	0.195157	0.212121	0.295918
Waist-to-hip ratio adjusted for BMI	rs521977	9.00E-19	0.533993	0.50505	0.421162
Height	rs4072910	1.00E-18	0.0484712	0.0858586	0.157143
Serum alkaline phosphatase levels	rs73196842	1.00E-18	0.751905	0.732323	0.856275
Inflammatory skin disease	rs6596086	2.00E-18	0.326305	0.368687	0.200405
Total cholesterol levels	rs2247056	2.00E-18	0.176141	0.161616	0.285425
Waist-to-hip ratio adjusted for BMI	rs2233956	3.00E-18	0.56275	0.535354	0.698381
Alzheimer's disease or family history of Alzheimer's disease	rs6656401	4.00E-18	0.587893	0.575758	0.693089
Cervical cancer	rs2516448	4.00E-18	0.317259	0.378788	0.427126
Emphysema distribution in smoking	rs13141641	6.00E-18	0.739669	0.737374	0.621951
HDL cholesterol levels x long total sleep time interaction (2df test)	rs2000813	6.00E-18	0.63213	0.661616	0.734818

Waist-to-hip ratio adjusted for BMI	rs592229	6.00E-18	0.13551	0.111111	0.260163
Chronic inflammatory diseases (ankylosing spondylitis, Crohn's disease, psoriasis, primary sclerosing cholangitis, ulcerative colitis) (pleiotropy)	rs925255	8.00E-18	0.471536	0.50505	0.348178
IgG monogalactosylation phenotypes (multivariate analysis)	rs11847263	1.00E-17	0.386088	0.39899	0.493902
Mitochondrial DNA levels	rs342293	1.00E-17	0.712292	0.691919	0.595142
Schizophrenia	rs7085104	1.00E-17	0.335426	0.393939	0.441057
Systolic blood pressure	rs185819	1.00E-17	0.454746	0.449495	0.591093
Waist-hip index	rs2233956	2.00E-17	0.56275	0.535354	0.698381
Electrocardiographic traits	rs7660702	3.00E-17	0.577608	0.616162	0.44332
Parkinson's disease	rs199515	3.00E-17	0.464537	0.449495	0.571138
Blood protein levels	rs687621	4.00E-17	0.650877	0.671717	0.753036
Height	rs10946808	4.00E-17	0.287209	0.308081	0.438776
A body shape index	rs630379	5.00E-17	0.0906701	0.0808081	0.210526
Vitamin B12 levels	rs492602	5.00E-17	0.476057	0.449495	0.609312
Interleukin-6 levels in HIV infection	rs4133213	6.00E-17	0.565055	0.565657	0.443089
Systolic blood pressure	rs932764	7.00E-17	0.256946	0.232323	0.390688
Apolipoprotein A1 levels	rs1446585	1.00E-16	0.520976	0.50505	0.693089
Cholesterol, total	rs492602	1.00E-16	0.476057	0.449495	0.609312
Crohn's disease	rs925255	1.00E-16	0.471536	0.50505	0.348178
Intelligence	rs1486091	1.00E-16	0.315664	0.313131	0.416327
Low density lipoprotein cholesterol levels	rs492602	1.00E-16	0.476057	0.449495	0.609312
Low density lipoprotein cholesterol levels	rs516246	1.00E-16	0.195157	0.212121	0.295918
Systolic blood pressure	rs6495122	1.00E-16	0.300596	0.5	0.495935
Ulcerative colitis	rs7134599	1.00E-16	0.290886	0.267677	0.396341
White blood cell count	rs342293	1.00E-16	0.712292	0.691919	0.595142
Asthma	rs2244012	2.00E-16	0.557859	0.530303	0.445122
Hip index	rs12449964	2.00E-16	0.489737	0.479798	0.382591
Pancreatic cancer	rs687289	2.00E-16	0.578966	0.555556	0.461538
Schizophrenia (MTAG)	rs7085104	2.00E-16	0.335426	0.393939	0.441057

Anorexia nervosa, attention-deficit/hyperactivity disorder, autism spectrum disorder, bipolar disorder, major depression, obsessive-compulsive disorder, schizophrenia, or Tourette syndrome (pleiotropy)	rs7085104	3.00E-16	0.335426	0.393939	0.441057
General risk tolerance (MTAG)	rs2047134	3.00E-16	0.399067	0.393939	0.265306
IgG digalactosylation phenotypes (multivariate analysis)	rs11847263	3.00E-16	0.386088	0.39899	0.493902
Serum metabolite levels	rs2403254	3.00E-16	0.29284	0.292929	0.188259
Waist-hip index	rs521977	3.00E-16	0.533993	0.50505	0.421162
Cardiovascular disease	rs943580	4.00E-16	0.709938	0.70202	0.563008
Lung cancer in ever smokers	rs2233956	5.00E-16	0.56275	0.535354	0.698381
Serum phosphate levels	rs2046210	5.00E-16	0.190511	0.166667	0.340081
HDL cholesterol levels x short total sleep time interaction (2df test)	rs2000813	6.00E-16	0.63213	0.661616	0.734818
Body fat percentage and HDL-C (pairwise)	rs3822072	8.00E-16	0.434077	0.479798	0.536437
IgA nephropathy	rs2856717	1.00E-15	0.403942	0.474747	0.50813
IgG galactosylation phenotypes (multivariate analysis)	rs11847263	1.00E-15	0.386088	0.39899	0.493902
Waist-hip index	rs592229	1.00E-15	0.13551	0.111111	0.260163
Asthma	rs204993	2.00E-15	0.504532	0.59596	0.623984
Blood pressure	rs1378942	2.00E-15	0.180921	0.136364	0.283673
Mean arterial pressure	rs6495122	2.00E-15	0.300596	0.5	0.495935
Platelet reactivity measurement (collagen-ADP)	rs687621	2.00E-15	0.650877	0.671717	0.753036
Triglycerides	rs2247056	2.00E-15	0.176141	0.161616	0.285425
Inflammatory bowel disease	rs925255	3.00E-15	0.471536	0.50505	0.348178
Alzheimer's disease or fasting insulin levels (pleiotropy)	rs6656401	4.00E-15	0.587893	0.575758	0.693089
Chronic obstructive pulmonary disease (severe)	rs13141641	4.00E-15	0.739669	0.737374	0.621951
Feeling worry	rs2269426	4.00E-15	0.24326	0.171717	0.1417
Hip circumference adjusted for BMI	rs592229	5.00E-15	0.13551	0.111111	0.260163
IgG sialylation phenotypes (multivariate analysis)	rs11847263	5.00E-15	0.386088	0.39899	0.493902
PR interval	rs13137008	5.00E-15	0.357905	0.333333	0.255102

IgG monosialylation phenotypes (multivariate analysis)	rs11847263	6.00E-15	0.386088	0.39899	0.493902
Liver enzyme levels	rs1780324	7.00E-15	0.705066	0.737374	0.506073
Cutaneous malignant melanoma	rs1056927	8.00E-15	0.113654	0.116162	0.278455
Gut microbiota abundance (phylum Actinobacteria id.400)	rs7570971	1.00E-14	0.283609	0.247475	0.392713
HDL cholesterol levels	rs1446585	1.00E-14	0.520976	0.50505	0.693089
Neurociticism	rs2269426	1.00E-14	0.24326	0.171717	0.1417
PR segment duration	rs13137008	1.00E-14	0.357905	0.333333	0.255102
Parkinson's disease	rs199533	1.00E-14	0.350345	0.373737	0.47551
Waist-to-hip ratio adjusted for BMI	rs630379	1.00E-14	0.0906701	0.0808081	0.210526
Alzheimer's disease	rs6656401	2.00E-14	0.587893	0.575758	0.693089
Systemic sclerosis	rs1378942	2.00E-14	0.180921	0.136364	0.283673
Serum uric acid levels	rs2437817	3.00E-14	0.591197	0.646465	0.477733
Alzheimer's disease	rs3818361	4.00E-14	0.717336	0.727273	0.566802
Post bronchodilator FEV1	rs13141641	4.00E-14	0.739669	0.737374	0.621951
Body mass index	rs9400239	5.00E-14	0.238693	0.237374	0.342857
Triglyceride levels	rs3822072	5.00E-14	0.434077	0.479798	0.536437
Type 1 diabetes	rs516246	5.00E-14	0.195157	0.212121	0.295918
Protein quantitative trait loci	rs778809	6.00E-14	0.632169	0.59596	0.491837
Retinol levels	rs1667255	6.00E-14	0.603034	0.611111	0.711382
Breast cancer	rs12493607	7.00E-14	0.356516	0.353535	0.251012
Human milk oligosaccharide concentration (fucodisialyllacto-N-hexaose)	rs516246	7.00E-14	0.195157	0.212121	0.295918
Inflammatory bowel disease	rs9358372	9.00E-14	0.193465	0.232323	0.303644
Serum metabolite levels	rs492602	9.00E-14	0.476057	0.449495	0.609312
Alzheimer's disease (late onset)	rs11771145	1.00E-13	0.504637	0.555556	0.390947
Cholesterol, total	rs7570971	1.00E-13	0.283609	0.247475	0.392713
Fat-free mass	rs9398171	1.00E-13	0.514964	0.5	0.617409
Inflammatory bowel disease	rs516246	1.00E-13	0.195157	0.212121	0.295918
LDL cholesterol levels x alcohol consumption (drinkers vs non-drinkers) interaction (2df)	rs7570971	1.00E-13	0.283609	0.247475	0.392713
Lung function (FEV1/FVC)	rs1028655	1.00E-13	0.720544	0.747475	0.833333

Parkinson's disease	rs329648	1.00E-13	0.359581	0.323232	0.256098
Platelet count	rs342296	1.00E-13	0.198514	0.191919	0.306911
Autoimmune traits	rs4713462	2.00E-13	0.26586	0.272727	0.373469
Blood protein levels	rs11753208	2.00E-13	0.530189	0.520202	0.632114
Graves' disease	rs3761959	2.00E-13	0.322011	0.318182	0.219512
Smoking initiation	rs7096169	2.00E-13	0.146111	0.141414	0.27439
Waist-hip index	rs630379	2.00E-13	0.0906701	0.0808081	0.210526
Coronary artery disease	rs644045	3.00E-13	0.460405	0.464646	0.352227
Waist-hip index	rs12449964	3.00E-13	0.489737	0.479798	0.382591
Waist-to-hip ratio adjusted for BMI (adjusted for smoking behaviour)	rs9991328	3.00E-13	0.230671	0.30303	0.346154
Creatinine levels	rs4805025	4.00E-13	0.29056	0.267677	0.184211
HDL cholesterol	rs1446585	4.00E-13	0.520976	0.50505	0.693089
High density lipoprotein cholesterol levels	rs9991328	4.00E-13	0.230671	0.30303	0.346154
Hip circumference adjusted for BMI	rs2244020	4.00E-13	0.35574	0.358586	0.461538
Neutrophil percentage of white cells	rs2072081	4.00E-13	0.316406	0.348485	0.427126
White blood cell count	rs2517510	4.00E-13	0.439649	0.454545	0.319106
Monocyte count	rs736227	5.00E-13	0.206377	0.191919	0.330612
Coronary artery disease	rs2072633	6.00E-13	0.130271	0.131313	0.257085
Heel bone mineral density	rs11712061	6.00E-13	0.443002	0.469697	0.544534
LDL cholesterol levels x alcohol consumption (regular vs non-regular drinkers) interaction (2df)	rs7570971	6.00E-13	0.283609	0.247475	0.392713
Lymphocyte percentage of white cells	rs2072081	6.00E-13	0.316406	0.348485	0.427126
Vitamin B12 levels	rs602662	6.00E-13	0.294849	0.242424	0.192308
Cold sores	rs885950	7.00E-13	0.473027	0.444444	0.345528
Graves' disease	rs4713693	7.00E-13	0.0934664	0.242424	0.24187
Psoriasis	rs492602	7.00E-13	0.476057	0.449495	0.609312
Total cholesterol levels	rs6495122	7.00E-13	0.300596	0.5	0.495935
Waist-to-hip ratio adjusted for BMI	rs12449964	7.00E-13	0.489737	0.479798	0.382591
Body mass index	rs261967	8.00E-13	0.300801	0.282828	0.189024
Estimated glomerular filtration rate	rs4805025	8.00E-13	0.29056	0.267677	0.184211

Fibroblast growth factor 23 levels	rs687289	1.00E-12	0.578966	0.555556	0.461538
Plateletcrit	rs6141755	1.00E-12	0.523044	0.510101	0.380567
Urinary metabolites (H-NMR features)	rs687289	1.00E-12	0.578966	0.555556	0.461538
A body shape index	rs2261033	2.00E-12	0.175818	0.181818	0.298781
Automobile speeding propensity	rs185819	2.00E-12	0.454746	0.449495	0.591093
Basal cell carcinoma	rs2153271	2.00E-12	0.684628	0.722222	0.802846
Blood protein levels in cardiovascular risk	rs492602	2.00E-12	0.476057	0.449495	0.609312
Breast cancer	rs3903072	2.00E-12	0.000755827	0.010101	0.0242915
Chronic obstructive pulmonary disease (moderate to severe)	rs13141641	2.00E-12	0.739669	0.737374	0.621951
Emphysema imaging phenotypes	rs13141641	2.00E-12	0.739669	0.737374	0.621951
Monocyte count	rs4954218	2.00E-12	0.585565	0.545455	0.461382
Non-melanoma skin cancer	rs2153271	2.00E-12	0.684628	0.722222	0.802846
Systemic lupus erythematosus	rs2301271	2.00E-12	0.608401	0.626263	0.714286
Waist-to-hip ratio adjusted for BMI	rs2261033	2.00E-12	0.175818	0.181818	0.298781
Blood protein levels	rs2853928	3.00E-12	0.0763136	0.156566	0.200405
High density lipoprotein cholesterol levels	rs3822072	3.00E-12	0.434077	0.479798	0.536437
Neuroticism	rs2269426	3.00E-12	0.24326	0.171717	0.1417
HDL cholesterol	rs3822072	4.00E-12	0.434077	0.479798	0.536437
Height	rs2247056	4.00E-12	0.176141	0.161616	0.285425
Mean arterial pressure	rs699	4.00E-12	0.551494	0.676768	0.691057
A body shape index	rs592229	5.00E-12	0.13551	0.111111	0.260163
Blood metabolite levels	rs6430553	6.00E-12	0.541194	0.535354	0.650826
Height	rs2523578	6.00E-12	0.472122	0.50505	0.597166
Height	rs6543146	6.00E-12	0.752343	0.80303	0.853659
Crohn's disease	rs281379	7.00E-12	0.34576	0.318182	0.447154
Heart rate	rs11154027	7.00E-12	0.296242	0.287879	0.178138
Mammographic density (dense area)	rs492602	7.00E-12	0.476057	0.449495	0.609312
Urate levels	rs2437817	7.00E-12	0.591197	0.646465	0.477733
Mean platelet volume	rs7317038	8.00E-12	0.303983	0.29798	0.20122

Birth weight	rs516246	9.00E-12	0.195157	0.212121	0.295918
Alzheimer's disease or family history of Alzheimer's disease	rs7810606	1.00E-11	0.54671	0.484848	0.653689
Electrocardiographic traits (multivariate)	rs7692808	1.00E-11	0.680172	0.681818	0.55668
Fasting insulin	rs3775380	1.00E-11	0.333318	0.348485	0.458678
IgG glycosylation	rs137686	1.00E-11	0.751394	0.661616	0.637652
Mean platelet volume	rs342296	1.00E-11	0.198514	0.191919	0.306911
Subcortical volume (MOSTest)	rs9398171	1.00E-11	0.514964	0.5	0.617409
Eosinophil percentage of white cells	rs6141755	2.00E-11	0.523044	0.510101	0.380567
Hip circumference adjusted for BMI	rs11855963	2.00E-11	0.216433	0.242424	0.317814
Hip circumference adjusted for BMI	rs1265093	2.00E-11	0.784199	0.782828	0.668699
Lung function (FEV1/FVC)	rs492602	2.00E-11	0.476057	0.449495	0.609312
Schizophrenia	rs9398171	2.00E-11	0.514964	0.5	0.617409
Waist-to-hip ratio adjusted for BMI	rs1265093	2.00E-11	0.784199	0.782828	0.668699
Waist circumference	rs9400239	2.00E-11	0.238693	0.237374	0.342857
Educational attainment	rs9393692	3.00E-11	0.765002	0.787879	0.651822
Male-pattern baldness	rs3850167	3.00E-11	0.102079	0.10101	0.208502
Waist-to-hip ratio adjusted for body mass index	rs9991328	3.00E-11	0.230671	0.30303	0.346154
Alzheimer's disease or family history of Alzheimer's disease	rs11771145	4.00E-11	0.504637	0.555556	0.390947
Depressive symptoms	rs2269426	4.00E-11	0.24326	0.171717	0.1417
Immature fraction of reticulocytes	rs7085104	4.00E-11	0.335426	0.393939	0.441057
Red blood cell count	rs2057053	4.00E-11	0.614436	0.60101	0.75813
Cognitive performance (MTAG)	rs4933677	5.00E-11	0.384238	0.333333	0.280488
Waist-hip index	rs2261033	5.00E-11	0.175818	0.181818	0.298781
Menarche (age at onset)	rs2836950	6.00E-11	0.45632	0.474747	0.560976
Body mass index	rs2836754	7.00E-11	0.314525	0.353535	0.206967
HDL cholesterol levels	rs3822072	7.00E-11	0.434077	0.479798	0.536437
Height	rs2261033	9.00E-11	0.175818	0.181818	0.298781
Height	rs537160	9.00E-11	0.220741	0.207071	0.355691
A body shape index	rs73198970	1.00E-10	0.14302	0.156566	0.243852

Body fat percentage and triglycerides (pairwise)	rs3822072	1.00E-10	0.434077	0.479798	0.536437
Cerebrospinal fluid biomarker levels	rs13437082	1.00E-10	0.599336	0.585859	0.487805
Eosinophil counts	rs4143832	1.00E-10	0.674802	0.676768	0.779352
High density lipoprotein cholesterol levels	rs28932178	1.00E-10	0.299674	0.308081	0.404858
Hip circumference adjusted for BMI	rs2256183	1.00E-10	0.316218	0.378788	0.427126
Intelligence (MTAG)	rs4261436	1.00E-10	0.493088	0.464646	0.367886
Pulse pressure	rs11154027	1.00E-10	0.296242	0.287879	0.178138
Rheumatoid arthritis	rs3761959	1.00E-10	0.322011	0.318182	0.219512
Smoking initiation (ever regular vs never regular) (MTAG)	rs1525164	1.00E-10	0.479093	0.429293	0.587045
Waist-hip index	rs1265093	1.00E-10	0.784199	0.782828	0.668699
Waist circumference adjusted for body mass index	rs73198970	1.00E-10	0.14302	0.156566	0.243852
A body shape index	rs2072633	2.00E-10	0.130271	0.131313	0.257085
Autism	rs4307059	2.00E-10	0.482239	0.409091	0.587398
Brain region volumes	rs2159676	2.00E-10	0.234207	0.277778	0.360324
Coronary artery disease or large artery stroke	rs12936587	2.00E-10	0.43639	0.409091	0.554878
Myopia (pathological)	rs16872571	2.00E-10	0.542234	0.520202	0.438776
Prostate cancer	rs10009409	2.00E-10	0.414284	0.429293	0.518293
Serum total protein level	rs73196842	2.00E-10	0.751905	0.732323	0.856275
Coronary artery disease	rs12936587	3.00E-10	0.43639	0.409091	0.554878
PR interval	rs7660702	3.00E-10	0.577608	0.616162	0.44332
Pulse pressure	rs932764	3.00E-10	0.256946	0.232323	0.390688
Coronary heart disease	rs12936587	4.00E-10	0.43639	0.409091	0.554878
Cutaneous lupus erythematosus	rs3130564	4.00E-10	0.613219	0.671717	0.733607
Digestive system disease (Barrett's esophagus and esophageal adenocarcinoma combined)	rs10419226	4.00E-10	0.314421	0.308081	0.194332
Freckling	rs2153271	4.00E-10	0.684628	0.722222	0.802846
Alzheimer's disease	rs11771145	5.00E-10	0.504637	0.555556	0.390947
Apolipoprotein A1 levels	rs3729856	5.00E-10	0.426002	0.434343	0.556017

Waist circumference adjusted for body mass index	rs2409694	5.00E-10	0.774587	0.752525	0.667347
Diastolic blood pressure	rs932764	6.00E-10	0.256946	0.232323	0.390688
Hemoglobin	rs2158007	6.00E-10	0.575455	0.530303	0.471074
IgG fucosylation phenotypes (multivariate analysis)	rs11847263	6.00E-10	0.386088	0.39899	0.493902
QT interval	rs938291	6.00E-10	0.355551	0.348485	0.518219
Adolescent idiopathic scoliosis	rs687621	7.00E-10	0.650877	0.671717	0.753036
LDL cholesterol levels in current drinkers	rs7570971	7.00E-10	0.283609	0.247475	0.392713
Hip circumference adjusted for BMI	rs3873388	8.00E-10	0.266219	0.257576	0.382114
Liver enzyme levels (gamma-glutamyl transferase)	rs516246	8.00E-10	0.195157	0.212121	0.295918
Problematic alcohol use	rs492602	8.00E-10	0.476057	0.449495	0.609312
Type 2 diabetes	rs261967	8.00E-10	0.300801	0.282828	0.189024
Hip circumference adjusted for BMI	rs2233956	9.00E-10	0.56275	0.535354	0.698381
Non-albumin protein levels	rs4954218	9.00E-10	0.585565	0.545455	0.461382
Non-albumin protein levels	rs73196842	9.00E-10	0.751905	0.732323	0.856275
Adolescent idiopathic scoliosis	rs7870976	1.00E-09	0.386764	0.40404	0.497967
BRCA1/2-negative high-risk breast cancer	rs2046210	1.00E-09	0.190511	0.166667	0.340081
Basophil percentage of white cells	rs2256183	1.00E-09	0.316218	0.378788	0.427126
Corneal structure	rs4954218	1.00E-09	0.585565	0.545455	0.461382
Diastolic blood pressure x smoking status (ever vs never) interaction (2df test)	rs2071550	1.00E-09	0.657921	0.661616	0.552632
Intelligence (MTAG)	rs1026997	1.00E-09	0.422297	0.383838	0.321862
Non-melanoma skin cancer	rs6510827	1.00E-09	0.574611	0.60101	0.455466
Peptic ulcer disease	rs687621	1.00E-09	0.650877	0.671717	0.753036
Serum alkaline phosphatase levels	rs3729856	1.00E-09	0.426002	0.434343	0.556017
Strenuous sports or other exercises	rs10946808	1.00E-09	0.287209	0.308081	0.438776
Triglyceride levels	rs73196842	1.00E-09	0.751905	0.732323	0.856275
Autism spectrum disorder, attention deficit-hyperactivity disorder, bipolar disorder, major	rs7914558	2.00E-09	0.462771	0.439394	0.330612

depressive disorder, and schizophrenia (combined)					
Generalized epilepsy	rs13026414	2.00E-09	0.388402	0.414141	0.263158
Hip circumference adjusted for BMI	rs12449964	2.00E-09	0.489737	0.479798	0.382591
Itch intensity from mosquito bite adjusted by bite size	rs9263475	2.00E-09	0.689329	0.656566	0.57085
Platelet count	rs397969	2.00E-09	0.396805	0.439394	0.538618
Prostate-specific antigen levels	rs3213764	2.00E-09	0.329329	0.353535	0.434694
Triglycerides	rs3822072	2.00E-09	0.434077	0.479798	0.536437
Type 2 diabetes	rs9991328	2.00E-09	0.230671	0.30303	0.346154
A body shape index	rs2233956	3.00E-09	0.56275	0.535354	0.698381
Alanine aminotransferase levels	rs2038648	3.00E-09	0.271646	0.292929	0.391393
Alanine aminotransferase levels	rs73196842	3.00E-09	0.751905	0.732323	0.856275
Childhood asthma with severe exacerbations	rs281379	3.00E-09	0.34576	0.318182	0.447154
Feeling miserable	rs17681615	3.00E-09	0.517715	0.489899	0.407787
Hip circumference adjusted for BMI	rs2409694	3.00E-09	0.774587	0.752525	0.667347
Hip circumference adjusted for BMI	rs4713462	3.00E-09	0.26586	0.272727	0.373469
BK polyomavirus VP1 antibody levels	rs492602	4.00E-09	0.476057	0.449495	0.609312
Diastolic blood pressure	rs6512586	4.00E-09	0.655194	0.641414	0.544534
Eosinophil counts	rs11055989	4.00E-09	0.303098	0.29798	0.412602
Family history of Alzheimer's disease	rs6656401	4.00E-09	0.587893	0.575758	0.693089
Hip circumference adjusted for BMI	rs2051549	4.00E-09	0.343306	0.373737	0.46473
Triglycerides	rs9991328	4.00E-09	0.230671	0.30303	0.346154
Alzheimer's disease (onset between ages 58 and 79)	rs3818361	5.00E-09	0.717336	0.727273	0.566802
Body mass index	rs7570971	5.00E-09	0.283609	0.247475	0.392713
Breast Cancer in BRCA1 mutation carriers	rs2046210	5.00E-09	0.190511	0.166667	0.340081
Hip index	rs767761	5.00E-09	0.487932	0.429293	0.37247
Chronic obstructive pulmonary disease-related biomarkers	rs1265093	6.00E-09	0.784199	0.782828	0.668699

Coronary artery disease	rs204993	6.00E-09	0.504532	0.59596	0.623984
Serum lipase activity	rs632111	6.00E-09	0.467145	0.489899	0.584711
Coffee consumption	rs6495122	7.00E-09	0.300596	0.5	0.495935
Type 2 diabetes	rs2244020	7.00E-09	0.35574	0.358586	0.461538
White blood cell count	rs3094212	7.00E-09	0.410905	0.469697	0.512195
Insulinoma-associated antigen 2 autoantibody levels in type 1 diabetes	rs7528684	8.00E-09	0.522883	0.565657	0.629098
Height	rs1560489	9.00E-09	0.627861	0.656566	0.755061
Hypertension	rs932764	9.00E-09	0.256946	0.232323	0.390688
Parental longevity (father's age at death or father's attained age)	rs3131621	9.00E-09	0.716931	0.707071	0.601626
Age-related macular degeneration	rs1999930	1.00E-08	0.680572	0.656566	0.572874
Body mass index	rs1446585	1.00E-08	0.520976	0.50505	0.693089
C-reactive protein levels	rs4705952	1.00E-08	0.510463	0.535354	0.395918
Coronary artery disease and LDL cholesterol levels (multivariate analysis)	rs7033354	1.00E-08	0.537138	0.560606	0.434694
Crohn's disease	rs9358372	1.00E-08	0.193465	0.232323	0.303644
Depressive symptoms	rs4713693	1.00E-08	0.0934664	0.242424	0.24187
Hip circumference adjusted for BMI	rs3094219	1.00E-08	0.726498	0.727273	0.611336
IgE levels	rs2858331	1.00E-08	0.569186	0.631313	0.467611
Major depressive disorder	rs6476606	1.00E-08	0.323485	0.282828	0.222672
Medication use (agents acting on the renin-angiotensin system)	rs516246	1.00E-08	0.195157	0.212121	0.295918
Neuroticism	rs4713693	1.00E-08	0.0934664	0.242424	0.24187
Neutrophil count	rs7155375	1.00E-08	0.221341	0.207071	0.359756
Retinal nerve fibre layer (RNFL) thickness	rs9398171	1.00E-08	0.514964	0.5	0.617409
Rheumatoid arthritis (ACPA-positive)	rs3761959	1.00E-08	0.322011	0.318182	0.219512
Systemic mastocytosis	rs1479010	1.00E-08	0.529217	0.565657	0.635628
Type 2 diabetes	rs687621	1.00E-08	0.650877	0.671717	0.753036
Coronary artery disease	rs699	2.00E-08	0.551494	0.676768	0.691057
Coronary artery disease and total cholesterol levels (multivariate analysis)	rs1378942	2.00E-08	0.180921	0.136364	0.283673

Coronary artery disease or ischemic stroke	rs12449964	2.00E-08	0.489737	0.479798	0.382591
Crohn's disease	rs504963	2.00E-08	0.563316	0.459596	0.435223
Endometriosis	rs644045	2.00E-08	0.460405	0.464646	0.352227
Eosinophil counts	rs7578035	2.00E-08	0.242955	0.262626	0.348178
Highest math class taken	rs73095857	2.00E-08	0.401719	0.469697	0.520576
Hip circumference adjusted for BMI	rs7741091	2.00E-08	0.419736	0.39899	0.548583
Infant length	rs592229	2.00E-08	0.13551	0.111111	0.260163
LDL cholesterol levels	rs7570971	2.00E-08	0.283609	0.247475	0.392713
Low density lipoprotein cholesterol levels	rs2247056	2.00E-08	0.176141	0.161616	0.285425
Monocyte count	rs687621	2.00E-08	0.650877	0.671717	0.753036
Psoriatic arthritis	rs2621322	2.00E-08	0.391338	0.39899	0.281377
Response to Dalcetrapib treatment in acute coronary syndrome	rs1967309	2.00E-08	0.354484	0.368687	0.45625
Schizophrenia	rs7914558	2.00E-08	0.462771	0.439394	0.330612
Triglycerides	rs492602	2.00E-08	0.476057	0.449495	0.609312
Triglycerides	rs516246	2.00E-08	0.195157	0.212121	0.295918
Vitiligo	rs16872571	2.00E-08	0.542234	0.520202	0.438776
Waist-to-hip ratio adjusted for BMI	rs4713460	2.00E-08	0.635488	0.631313	0.754065
Waist-to-hip ratio adjusted for BMI x sex x age interaction (4df test)	rs9991328	2.00E-08	0.230671	0.30303	0.346154
Alcohol use disorder (total score)	rs492602	3.00E-08	0.476057	0.449495	0.609312
C-reactive protein levels or LDL-cholesterol levels (pleiotropy)	rs2287921	3.00E-08	0.222333	0.212121	0.109312
Chronic bronchitis and chronic obstructive pulmonary disease	rs12692398	3.00E-08	0.570573	0.565657	0.674797
Depression (broad)	rs6483414	3.00E-08	0.311457	0.272727	0.44332
Distal/Left-sided colorectal cancer	rs1446585	3.00E-08	0.520976	0.50505	0.693089
HTLV-1 associated myelopathy	rs2523554	3.00E-08	0.514123	0.459596	0.384146
Height	rs185819	3.00E-08	0.454746	0.449495	0.591093
Hip circumference	rs12936587	3.00E-08	0.43639	0.409091	0.554878
Hip circumference adjusted for BMI	rs241429	3.00E-08	0.212	0.232323	0.313008

Neuroticism conditioned on average household income before tax (multi-trait conditioning and joint analysis)	rs1990277	3.00E-08	0.338987	0.333333	0.230453
Schizophrenia (MTAG)	rs2192932	3.00E-08	0.419716	0.419192	0.53125
Allergic sensitization	rs6932730	4.00E-08	0.639432	0.666667	0.518293
Appendicular lean mass	rs261967	4.00E-08	0.300801	0.282828	0.189024
IgE levels	rs2040704	4.00E-08	0.646431	0.712121	0.512146
Immunoglobulin A vasculitis	rs2858331	4.00E-08	0.569186	0.631313	0.467611
Parental longevity (father's attained age)	rs3131621	4.00E-08	0.716931	0.707071	0.601626
Response to antipsychotic treatment	rs17382202	4.00E-08	0.644324	0.656566	0.759184
Weight	rs1589163	4.00E-08	0.367304	0.363636	0.487552
White blood cell count (lymphocyte)	rs2249742	4.00E-08	0.580392	0.575758	0.692308
Alzheimer's disease or family history of Alzheimer's disease	rs12036785	5.00E-08	0.698751	0.676768	0.593496
Breast cancer	rs199533	5.00E-08	0.350345	0.373737	0.47551
Cognitive performance (MTAG)	rs10513799	5.00E-08	0.341764	0.338384	0.475709
Diastolic blood pressure	rs687621	5.00E-08	0.650877	0.671717	0.753036
Diastolic blood pressure x smoking status (current vs non-current) interaction (2df test)	rs2071550	5.00E-08	0.657921	0.661616	0.552632
Height	rs13437082	5.00E-08	0.599336	0.585859	0.487805
Pediatric autoimmune diseases	rs602662	5.00E-08	0.294849	0.242424	0.192308
Pubertal anthropometrics	rs281379	5.00E-08	0.34576	0.318182	0.447154
Systemic lupus erythematosus	rs422544	5.00E-08	0.573779	0.555556	0.442387
AIDS progression	rs10484554	6.00E-08	0.677902	0.707071	0.558704
Barrett's esophagus	rs10419226	6.00E-08	0.314421	0.308081	0.194332
Triglyceride levels	rs17124780	6.00E-08	0.663985	0.69697	0.550607
Smoking status	rs7580488	8.00E-08	0.400652	0.358586	0.561475
Parental longevity (combined parental attained age, Martingale residuals)	rs3131621	9.00E-08	0.716931	0.707071	0.601626
Vitiligo	rs6510827	9.00E-08	0.574611	0.60101	0.455466
Body mass index	rs2108978	1.00E-07	0.859481	0.843434	0.756098
Breastfeeding duration	rs6950451	1.00E-07	0.395306	0.363636	0.497976

Waist-to-hip ratio adjusted for BMI (joint analysis main effects and smoking interaction)	rs9991328	1.00E-07	0.230671	0.30303	0.346154
Asthma	rs2040704	2.00E-07	0.646431	0.712121	0.512146
LDL cholesterol levels	rs492602	2.00E-07	0.476057	0.449495	0.609312
Vitamin B levels in ischemic stroke	rs492602	2.00E-07	0.476057	0.449495	0.609312
Caffeine consumption	rs2472304	3.00E-07	0.433325	0.409091	0.317814
Depression (broad)	rs537160	3.00E-07	0.220741	0.207071	0.355691
Height	rs7741091	3.00E-07	0.419736	0.39899	0.548583
Lupus nephritis in systemic lupus erythematosus	rs2647012	3.00E-07	0.414683	0.459596	0.311741
Non-Hodgkin's lymphoma	rs2858331	3.00E-07	0.569186	0.631313	0.467611
Platelet aggregation	rs342293	3.00E-07	0.712292	0.691919	0.595142
Post bronchodilator FEV1/FVC ratio in COPD	rs13141641	3.00E-07	0.739669	0.737374	0.621951
Cancer (pleiotropy)	rs199533	4.00E-07	0.350345	0.373737	0.47551
Perceived unattractiveness to mosquitoes	rs521977	4.00E-07	0.533993	0.50505	0.421162
Crohn's disease	rs2836754	5.00E-07	0.314525	0.353535	0.206967
HTLV-1 associated myelopathy	rs2647012	5.00E-07	0.414683	0.459596	0.311741
Caffeine consumption	rs6495122	6.00E-07	0.300596	0.5	0.495935
Adolescent idiopathic scoliosis	rs2830622	7.00E-07	0.378322	0.353535	0.495935
Esophageal adenocarcinoma	rs10419226	8.00E-07	0.314421	0.308081	0.194332
Neuroticism	rs859767	8.00E-07	0.366869	0.323232	0.485772
Select biomarker traits	rs746961	8.00E-07	0.26156	0.267677	0.376543
Itch intensity from mosquito bite adjusted by bite size	rs521977	9.00E-07	0.533993	0.50505	0.421162
Vitamin B levels in ischemic stroke	rs2287921	9.00E-07	0.222333	0.212121	0.109312
Colorectal cancer	rs4954585	1.00E-06	0.8265	0.848485	0.718623
Depression in response to interferon-based therapy in chronic hepatitis C	rs2779180	1.00E-06	0.782793	0.818182	0.635246
Family history of Alzheimer's disease	rs7810606	1.00E-06	0.54671	0.484848	0.653689
Gut microbiota (bacterial taxa, rank normal transformation method)	rs7570971	1.00E-06	0.283609	0.247475	0.392713
Maternal history of Alzheimer's disease	rs6656401	1.00E-06	0.587893	0.575758	0.693089

Multiple sclerosis and LDL levels (pleiotropy)	rs3761959	1.00E-06	0.322011	0.318182	0.219512
Psoriasis	rs9305556	1.00E-06	0.735419	0.742424	0.619342
Rosacea symptom severity	rs6598858	1.00E-06	0.442464	0.40404	0.548781
Tendinopathy	rs11154027	1.00E-06	0.296242	0.287879	0.178138
Waist-to-hip ratio adjusted for BMI in non-smokers	rs9991328	1.00E-06	0.230671	0.30303	0.346154
BMI (adjusted for smoking behaviour)	rs9400239	2.00E-06	0.238693	0.237374	0.342857
Cognitive performance	rs6739054	2.00E-06	0.438626	0.414141	0.548583
Coronary artery disease	rs2000813	2.00E-06	0.63213	0.661616	0.734818
Menarche (age at onset)	rs7114000	2.00E-06	0.326527	0.353535	0.436735
Methotrexate-induced interstitial lung disease in rheumatoid arthritis	rs9299346	2.00E-06	0.269649	0.272727	0.162602
Nicotine dependence	rs2836823	2.00E-06	0.38668	0.414141	0.268293
Obesity-related traits	rs2963826	2.00E-06	0.458532	0.474747	0.346154
Obesity-related traits	rs516246	2.00E-06	0.195157	0.212121	0.295918
Protein quantitative trait loci	rs241453	2.00E-06	0.799663	0.787879	0.676829
Telomere length	rs4452212	2.00E-06	0.212908	0.217172	0.317814
Waist-to-hip ratio adjusted for BMI (age <50)	rs9991328	2.00E-06	0.230671	0.30303	0.346154
Alcohol dependence or chronic alcoholic pancreatitis or alcohol-related liver cirrhosis	rs454510	3.00E-06	0.814674	0.858586	0.710526
Bipolar disorder	rs2287921	3.00E-06	0.222333	0.212121	0.109312
Bipolar disorder	rs7578035	3.00E-06	0.242955	0.262626	0.348178
Chronic bronchitis and chronic obstructive pulmonary disease	rs13141641	3.00E-06	0.739669	0.737374	0.621951
Cleft palate	rs228218	3.00E-06	0.454983	0.50505	0.346311
Eosinophil counts	rs2269426	3.00E-06	0.24326	0.171717	0.1417
Height	rs6670655	3.00E-06	0.567812	0.535354	0.45935
Mosquito bite size	rs806795	3.00E-06	0.71534	0.661616	0.59919
Multiple sclerosis	rs3761959	3.00E-06	0.322011	0.318182	0.219512
Number of children	rs12408989	3.00E-06	0.459509	0.484848	0.566802
Obesity-related traits	rs1867485	3.00E-06	0.451968	0.515152	0.558704
Scarlet fever	rs9296042	3.00E-06	0.22337	0.136364	0.347917

Triglyceride levels	rs7033354	3.00E-06	0.537138	0.560606	0.434694
Endometriosis	rs2929986	4.00E-06	0.146745	0.146465	0.247967
Epithelial ovarian cancer	rs243172	4.00E-06	0.3212	0.373737	0.42449
Height	rs27855	4.00E-06	0.445924	0.459596	0.591463
IgG glycosylation	rs592229	4.00E-06	0.13551	0.111111	0.260163
Pursuit maintenance gain	rs35791980	4.00E-06	0.470285	0.469697	0.574899
Sleep-related phenotypes	rs10492604	4.00E-06	0.600841	0.636364	0.495951
Thiazide-induced adverse metabolic effects in hypertensive patients	rs427576	4.00E-06	0.616316	0.712121	0.479592
Lymphoma	rs7453920	5.00E-06	0.375118	0.39899	0.483607
Mean arterial pressure	rs11169571	5.00E-06	0.331845	0.353535	0.445344
Neuropathologic traits (pleiotropy)	rs6540796	5.00E-06	0.118187	0.136364	0.219512
Systemic lupus erythematosus	rs1378942	5.00E-06	0.180921	0.136364	0.283673
Coronary heart disease	rs11650066	6.00E-06	0.675842	0.626263	0.567347
Gut microbiota relative abundance (unclassified genus belonging to family Ruminococcaceae)	rs6976603	6.00E-06	0.327252	0.328283	0.224696
Non-alcoholic fatty liver disease histology (other)	rs6027755	6.00E-06	0.104528	0.0959596	0.265182
Persistent hepatitis B virus infection	rs7453920	6.00E-06	0.375118	0.39899	0.483607
Blood protein levels	rs7102266	7.00E-06	0.393503	0.429293	0.29065
D-dimer levels	rs687621	7.00E-06	0.650877	0.671717	0.753036
IgG glycosylation	rs6064045	7.00E-06	0.517208	0.479798	0.396761
Metabolite levels	rs10496767	7.00E-06	0.502629	0.550505	0.389344
Number of pregnancies	rs12408989	7.00E-06	0.459509	0.484848	0.566802
Adverse response to chemotherapy (neutropenia/leucopenia) (cyclophosphamide)	rs3745571	8.00E-06	0.203549	0.191919	0.330612
Alanine aminotransferase levels in non-alcoholic fatty liver disease	rs16997877	8.00E-06	0.221097	0.207071	0.352227
Distal colorectal cancer	rs1446585	8.00E-06	0.520976	0.50505	0.693089
Energy expenditure (24h)	rs11117784	8.00E-06	0.466254	0.489899	0.352227
HDL cholesterol levels x alcohol consumption (regular vs non-regular drinkers) interaction (2df)	rs7570971	8.00E-06	0.283609	0.247475	0.392713

Hypertension	rs1378942	8.00E-06	0.180921	0.136364	0.283673
Multiple sclerosis	rs1841770	8.00E-06	0.168922	0.191919	0.27551
Systemic lupus erythematosus	rs2647012	8.00E-06	0.414683	0.459596	0.311741
Anorexia nervosa	rs28441017	9.00E-06	0.474007	0.459596	0.629098
Colorectal cancer	rs651907	9.00E-06	0.387515	0.40404	0.487805
IgG glycosylation	rs10877839	9.00E-06	0.630269	0.671717	0.516194
Metabolite levels	rs10066976	9.00E-06	0.500461	0.489899	0.601215
Quantitative traits	rs1874326	9.00E-06	0.0924628	0.106061	0.27439
Superior frontal gyrus grey matter volume	rs2623384	9.00E-06	0.506663	0.555556	0.390688
Ischemic stroke (cardioembolic)	rs938291	1.00E-05	0.355551	0.348485	0.518219

References

1. Rotar O, Moguchaia E, Boyarinova M, Kolesova E, Khromova N, Freylikhman O, et al. Seventy years after the siege of Leningrad: does early life famine still affect cardiovascular risk and aging? J Hypertens. 2015;33: 1772–9; discussion 1779.
2. Browning BL, Zhou Y, Browning SR. A One-Penny Imputed Genome from Next-Generation Reference Panels. Am J Hum Genet. 2018;103: 338–348.
3. Purcell S, Neale B, Todd-Brown K, Thomas L, Ferreira MAR, Bender D, et al. PLINK: a tool set for whole-genome association and population-based linkage analyses. Am J Hum Genet. 2007;81: 559–575.
4. Danecek P, Bonfield JK, Liddle J, Marshall J, Ohan V, Pollard MO, et al. Twelve years of SAMtools and BCFtools. Gigascience. 2021;10. doi:10.1093/gigascience/giab008
5. Consortium T 1000 GP, The 1000 Genomes Project Consortium. A global reference for human genetic variation. Nature. 2015. pp. 68–74. doi:10.1038/nature15393
6. Bergström A, McCarthy SA, Hui R, Almarri MA, Ayub Q, Danecek P, et al. Insights into human genetic variation and population history from 929 diverse genomes. Science. 2020;367. doi:10.1126/science.aay5012
7. McCarthy S, Das S, Kretzschmar W, Delaneau O, Wood AR, Teumer A, et al. A reference panel of 64,976 haplotypes for genotype imputation. Nat Genet. 2016;48: 1279–1283.
8. Choi SW, Mak TS-H, O’Reilly PF. Tutorial: a guide to performing polygenic risk score analyses. Nat Protoc. 2020;15: 2759–2772.
9. Rowan TN, Hoff JL, Crum TE, Taylor JF, Schnabel RD, Decker JE. A multi-breed reference panel and additional rare variants maximize imputation accuracy in cattle. Genet Sel Evol. 2019;51: 77.
10. Ramnarine S, Zhang J, Chen L-S, Culverhouse R, Duan W, Hancock DB, et al.

When Does Choice of Accuracy Measure Alter Imputation Accuracy Assessments? PLoS One. 2015;10: e0137601.

11. Machiela MJ, Chen C, Liang L, Diver WR, Stevens VL, Tsilidis KK, et al. One thousand genomes imputation in the National Cancer Institute Breast and Prostate Cancer Cohort Consortium aggressive prostate cancer genome-wide association study. Prostate. 2013;73: 677–689.
12. Li L, Huang P, Sun X, Wang S, Xu M, Liu S, et al. The ChinaMAP reference panel for the accurate genotype imputation in Chinese populations. Cell Res. 2021;31: 1308–1310.
13. Charon C, Allodji R, Meyer V, Deleuze J-F. Impact of pre- and post-variant filtration strategies on imputation. Sci Rep. 2021;11: 6214.
14. Wright S. THE GENETICAL STRUCTURE OF POPULATIONS. Annals of Eugenics. 1949. pp. 323–354. doi:10.1111/j.1469-1809.1949.tb02451.x
15. Danecek P, Auton A, Abecasis G, Albers CA, Banks E, DePristo MA, et al. The variant call format and VCFtools. Bioinformatics. 2011;27: 2156–2158.
16. Pagani L, Lawson DJ, Jagoda E, Mörseburg A, Eriksson A, Mitt M, et al. Genomic analyses inform on migration events during the peopling of Eurasia. Nature. 2016;538: 238–242.
